# Musculoskeletal pain and sedentary behaviour in occupational and non-occupational settings: a systematic review with meta-analysis

**DOI:** 10.1186/s12966-021-01191-y

**Published:** 2021-12-13

**Authors:** Francis Q. S. Dzakpasu, Alison Carver, Christian J. Brakenridge, Flavia Cicuttini, Donna M. Urquhart, Neville Owen, David W. Dunstan

**Affiliations:** 1 Mary MacKillop Institute of Health Research, Australian Catholic University, Melbourne, VIC Australia; 2 Physical Activity Laboratory, Baker Heart and Diabetes Institute, Melbourne, VIC Australia; 3 Behavioural Epidemiology Laboratory, Baker Heart and Diabetes Institute, Melbourne, VIC Australia; 4 Central Clinical School/Department of Epidemiology and Preventive Medicine, Faculty of Medicine, Nursing and Health Sciences, Monash University, Melbourne, VIC Australia; 5 Centre for Urban Transitions, Swinburne University of Technology, Melbourne, VIC Australia

**Keywords:** Sedentary behaviour (SB), Occupational, Non-occupational, Workplace sitting, Self-reported, Device-measured, Computer time, Vehicle time, Musculoskeletal pain (MSP) conditions

## Abstract

**Background:**

Sedentary behaviour (SB; time spent sitting) is associated with musculoskeletal pain (MSP) conditions; however, no prior systematic review has examined these associations according to SB domains. We synthesised evidence on occupational and non-occupational SB and MSP conditions.

**Methods:**

Guided by a PRISMA protocol, eight databases (MEDLINE, CINAHL, PsycINFO, Web of Science, Scopus, Cochrane Library, SPORTDiscus, and AMED) and three grey literature sources (Google Scholar, WorldChat, and Trove) were searched (January 1, 2000, to March 17, 2021) for original quantitative studies of adults ≥ 18 years. Clinical-condition studies were excluded. Studies’ risk of bias was assessed using the QualSyst checklist. For meta-analyses, random effect inverse-variance pooled effect size was estimated; otherwise, best-evidence synthesis was used for narrative review.

**Results:**

Of 178 potentially-eligible studies, 79 were included [24 general population; 55 occupational (incuding15 experimental/intervention)]; 56 studies were of high quality, with scores > 0.75. Data for 26 were meta-synthesised. For cross-sectional studies of non-occupational SB, meta-analysis showed full-day SB to be associated with low back pain [LBP – OR = 1.19(1.03 – 1.38)]. Narrative synthesis found full-day SB associations with knee pain, arthritis, and general MSP, but the evidence was insufficient on associations with neck/shoulder pain, hip pain, and upper extremities pain. Evidence of prospective associations of full-day SB with MSP conditions was insufficient. Also, there was insufficient evidence on both cross-sectional and prospective associations between leisure-time SB and MSP conditions. For occupational SB, cross-sectional studies meta-analysed indicated associations of self-reported workplace sitting with LBP [OR = 1.47(1.12 – 1.92)] and neck/shoulder pain [OR = 1.73(1.46 – 2.03)], but not with extremities pain [OR = 1.17(0.65 – 2.11)]. Best-evidence synthesis identified inconsistent findings on cross-sectional association and a probable negative prospective association of device-measured workplace sitting with LBP-intensity in tradespeople. There was cross-sectional evidence on the association of computer time with neck/shoulder pain, but insufficient evidence for LBP and general MSP. Experimental/intervention evidence indicated reduced LBP, neck/shoulder pain, and general MSP with reducing workplace sitting.

**Conclusions:**

We found cross-sectional associations of occupational and non-occupational SB with MSP conditions, with occupational SB associations being occupation dependent, however, reverse causality bias cannot be ruled out. While prospective evidence was inconclusive, reducing workplace sitting was associated with reduced MSP conditions. Future studies should emphasise prospective analyses and examining potential interactions with chronic diseases.

**Protocol registration:**

PROSPERO ID #CRD42020166412 (Amended to limit the scope)

**Supplementary Information:**

The online version contains supplementary material available at 10.1186/s12966-021-01191-y.

## Background

The burden of musculoskeletal pain (MSP) conditions has increased in recent decades, contributing to substantial health care costs [1]. According to 2019 Global Burden of Disease (GBD) estimates, age-standardised disability-adjusted life years attributable to MSP conditions excluding low back pain (LBP) increased from 1990 to 2019 by some 30.7 percentage points [2]; whereas the 2017 GDB report ranked LBP as the second-highest contributor to years lived with disability [3]. The prevalence of MSP conditions has increased in parallel with the rising burden of chronic disease and is most pronounced in those with multi-morbidities [3, 4]. Also, MSP can substantially limit mobility and engagement in regular physical activity, thereby predisposing to increased risk of other chronic conditions [3].

The biological mechanisms contributing to MSP conditions are heterogeneous; nonetheless, obesity, static working postures, physical inactivity, smoking, and aging, as well as cardiometabolic and systemic inflammation, are some factors identified to increase the prevalence of MSP [5, 6]. While there is convincing evidence of beneficial associations of physical activity with outcomes related to MSP conditions [7, 8] there is an additional element to consider in this nexus – sedentary behaviour (SB). Defined as time spent in sitting and/or reclining postures during waking hours, with energy expenditure less than 1.5 metabolic equivalents (METs) [9] – SB is associated with increased risk and unfavourable outcomes of chronic diseases, including cardiovascular disease, metabolic disorders, musculoskeletal diseases, and some cancers, as well as all-cause mortality [10, 11]. Intervention trials have shown that reducing sitting time can result in modest improvements in some biomarkers of health risk [12, 13]. From a population health perspective, excessive time spent sitting is common among older adults, especially in those with co-morbidities such as cardiovascular and metabolic disorders [14, 15].

Epidemiological evidence indicates higher volumes of SB are associated with several MSP conditions, including osteoarthritis, back pain, and neck/shoulder pain [16, 17]. Some of these findings are from low-level evidence cross-sectional studies and there could be potential reverse causality bias [16]; inferring a causal relationship between SB and MSP may therefore be problematic as pain and chronic disease could predispose to engagement in excessive SB [18]. There is, however, an inconsistent body of evidence of associations of SB with MSP conditions and related outcomes from high-level evidence-based studies [19, 20]. Some previous systematic reviews of studies including higher-level study designs have reported no associations of SB with the prevalence of some MSP conditions [19–24], whereas others have reported either positive [20, 25] or negative [26] associations with some MSP-related outcomes such as pain intensity. Methodological differences and limitations within the individual studies reviewed in these systematic reviews could impact the quality of evidence and comparability of these reviews as some of the studies were based on self-reported and surrogate estimates of SB which increases the risk of bias [19, 21, 22, 24, 27]. The emergence of evidence on device-measured SB, especially from studies using the ActiGraph and activPAL devices has improved the quality of SB evidence in recent research outputs [25–27].

There could be other reasons for the equivocal associations, including factors related to the influence of the specific domains of SB (e.g., work, transport, domestic) and the relative exposure of the studied population. This perspective suggests potential contributions of different domains of SB to the risk of adverse health outcomes, which may differ from the effects of total full-day SB [28–30]. Moreover, evidence on differences in health effects of different SB domains has been identified as a key knowledge gap by the 2020 World Health Organisation (WHO) physical activity and SB guidelines development group [31]. Existing systematic reviews have not identified differences according to domains in the associations of SB with MSP conditions.

This distinction is important, partly because, most working adults accumulate SB in both occupational and non-occupational settings. That said, SB could predispose to MSP conditions in certain occupational groups such as desk-based workers who commonly engage in a prolonged sitting [32, 33]. In this context, interventions to reduce prolonged workplace sitting time by breaking up sitting with standing and/or light walking have shown beneficial associations with a reduction in MSP or musculoskeletal system discomfort among desk-based workers [34, 35]. Thus, SB associations may also reflect plausible biomechanical or biological pathways explaining MSP conditions in those exposed to prolonged static sitting postures [36–38]. Paradoxically, however, in occupational groups such as tradespeople who engage in more labour-intensive manual work, SB may be a protective behaviour against MSP conditions and other chronic diseases [39–41].

We conducted a systematic review to examine evidence on the associations of SB with MSP conditions in observational and experimental/intervention studies of adults. Specifically, we examined and synthesised evidence separately for associations of SB with MSP conditions in the occupational and non-occupational SB domains.

## Methods

### Review design

We used a standard Preferred Reporting Items for Systematic Reviews and Meta-Analyses (PRISMA) guidelines-based pre-designed protocol (PROSPERO ID: CRD42020166412 – amended to limit the scope of the review) to ensure a transparent review [42, 43]. The a priori research question and search strategy were formulated according to the Population, Intervention, Control/Comparison, and Outcome (PICO) framework [44] to enhance search precision and ensure extensive data extraction to be representative and unbiased [45]. The research question was: *What are the associations of occupational and non-occupational SB with MSP conditions in adults?*

### Search strategy

Using a comprehensive search strategy, search terms were identified and combined using Boolean operators to search the following electronic databases: MEDLINE Complete, CINAHL Complete, PsycINFO, Web of Science, Scopus, Cochrane Library, SPORTDiscus, and AMED. Additionally, three online grey literature databases, including Google Scholar, WorldChat, and Trove, were searched to also identify non-peer-reviewed studies to help to minimise publication bias [46]. The search was conducted by one reviewer, for consistency, with the guidance of a librarian (Australian Catholic University, Melbourne) initially on January 5, 2020; and, further updated on November 1, 2020, and March 17, 2021. The search filter was set to limit search results to studies published from January 1, 2000, onwards. This timeframe was chosen because the field of SB is relatively new, the early definitive papers were published at the beginning of this period, and SB research output has grown significantly over the past two decades [9].

The search terms format, guided by the PICO framework, included keywords, terms, and phrases related to SB (Exposure/Intervention); MSP conditions (Outcome); and adults (Population). The search was optimized by adding to the search string, newly identified key terms that consistently appear in titles and abstracts of retrieved studies during the search [47]. A supplementary file (Supplementary Table 1: Search key terms and strings strategy – A sample Medline database search syntax) describing the comprehensive search term framework is attached.

### Study eligibility and selection

#### Inclusion and exclusion criteria

The selection of eligible studies was based on pre-determined inclusion and exclusion criteria. The reviewed studies satisfied all the criteria below:

- An original quantitative study involving either an observational or intervention/experimental design. This included cross-sectional, case–control studies, and prospective studies, as well as randomized controlled trials (RCTs) and non-randomized experimental study designs.
- The study was conducted in adults aged 18 years or older and examined relationships between SB (the exposure of interest) and MSP conditions (the outcome of interest).
- The study included a measure of any kind of MSP condition, including inflammatory and non-inflammatory MSP conditions such as back pain, joint/osteoarthritis, and pain in extremities (except for pain attributable, acutely or recently, to trauma). Autoimmune-related MSP conditions, for example, rheumatoid arthritis and fibromyalgia were not included in this review because the pathophysiology of these conditions is mainly attributable to the processes and progression of specific clinical disease entities with autoimmune causations. Some studies did not measure a specific type of MSP condition but produced a composite measure of MSP conditions. Those that measured arthritis but excluded fibromyalgia were considered for inclusion because the majority of reported cases of arthritis are likely to be osteoarthritis rather than rheumatoid arthritis. There is no universally accepted measure for MSP conditions; therefore, any acceptable measures described in studies provided the basis for considering studies to be appropriately inclusive of MSP conditions.
- The study clearly defined or stated the measure of SB. Specifically, the study reported a self-report measure or device-based measure of occupational or non-occupational SB. This included population-based or occupational/workgroup cohort studies that measured SB exposures that aligned with the focus of our review.

Studies were excluded if they met any of the criteria described below:

- all qualitative studies and those quantitative studies involving children and adolescent populations aged below 18 years;
- studies that did not appropriately define SB; those that used proxy estimates, such as “less active”, “inactive” or “does not engage in physical activities”; those that did not make a clear distinction between SB and physical inactivity and included these as overlapping behaviours or used these terms interchangeably;
- studies that focused on SB as an outcome but did not explicitly examine the relationship of SB with MSP conditions; studies that focused only on the relationship between physical activity and MSP conditions;
- studies conducted exclusively in clinical groups with existing clinically diagnosed MSP conditions, e.g., knee osteoarthritis patients that focused on symptom severity as outcome measures;
- opinion or perspective articles, conference papers, editorials, newsletters, and review studies, however, the reference lists of some literature reviews on a similar topic were hand-searched for relevant studies;
- studies published in languages other than English.

#### Screening and selection process

**Fig. 1:**
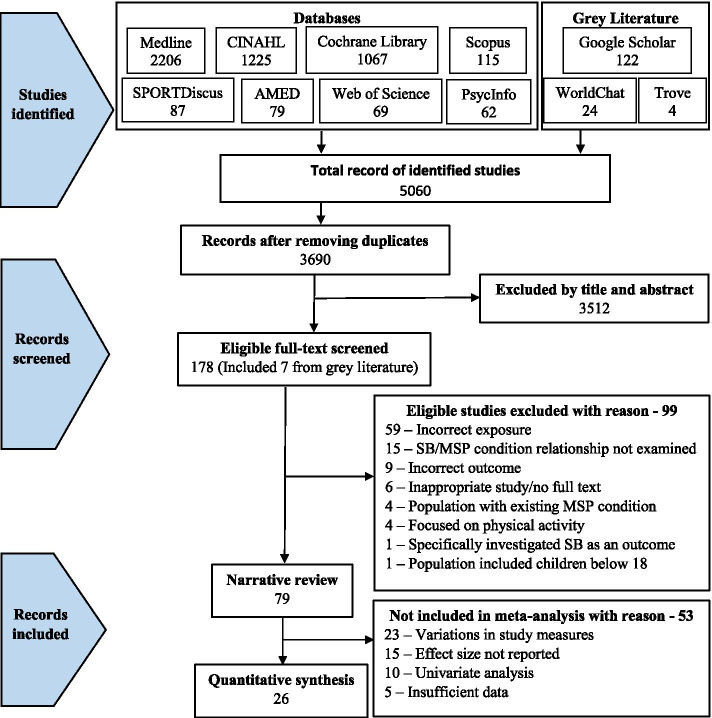
PRISMA flow diagram of the studies record

A two-stage approach was used to process all identified studies before arriving at the final set of studies for inclusion in this review. First, the reviewer (FD), exported all the retrieved studies into Endnote reference manager software [48], checked and removed duplicate studies. The refined list of studies was exported into collaboration-supported Rayyan systematic review software [49] for screening. One reviewer (FD) initially screened and removed irrelevant studies by title and abstract according to our inclusion and exclusion criteria, but where there was uncertainty regarding inclusion, such studies were considered in stage two screening. The second stage involved retrieval of full-text articles of retained studies, and two reviewers (FD and CB), independently read and assessed the full-text articles for inclusion. Disparities were discussed and resolved among the two reviewers; however, when uncertainty remained, they consulted with three senior reviewers (AC, NO and DD). Records of retained studies as well as reasons for exclusion (at stage two) were documented using a PRISMA flowchart (Fig. 1).

### Data extraction

A pre-designed data extraction form was used to organise relevant information from the studies reviewed, to ensure data quality, and to minimise errors [50]. Reviewer FD extracted data from all the studies, and this was verified independently by CB. The verification process involved the comparison of data extracted by CB from randomly selected studies (not less than 20%) with the extracts of FD [51]. Disagreements were resolved harmoniously. Extracted data included:

- Descriptive details – study title, author name, year of publication, place of study, study aim
- Study design – cross-sectional, case–control, prospective, experiment/RCT/non-RCT
- Study population – population-based, occupational/workgroup cohort
- Sample size
- Demographic information of study participants – e.g., gender, mean age or age range, and BMI.
- SB and measures – occupational SB, non-occupational SB, self-report and objective measures.
- Outcome variables and measures – MSP conditions, e.g., back pain, neck/shoulder pain, osteoarthritis, and extremities pain.
- Intervention/experiment detail (when applicable) – type, duration, assessment point(s), effect size, etc.
- Other relevant data relating to the MSP condition outcomes and their measures – e.g., pain intensity and disability.

### Study quality assessment

Quality assessment for the included studies was undertaken (independently by two reviewers) using the quantitative checklist of QualSyst (Standard Quality Assessment Criteria for Evaluating Primary Research Papers from a Variety of Fields) [52]. Briefly, the quantitative QualSyst checklist is scored on 14 criteria as either “YES = 2”, “PARTIAL = 1”, “NO = 0” or “NOT APPLICABLE” (N/A) depending on the extent to which each criterion item is satisfied by the study report. Items marked ‘N/A’ were excluded from the computation of the QualSyst summary score. For each paper, a summary score was computed by summing scores across items and dividing this by the maximum possible score for all relevant items [i.e., 28 – (number of ‘N/A’ items × 2)] [52]. Disparities in the assessments were discussed and resolved between the assessors, and if required, the three senior reviewers arbitrated. Note, however, that the quality assessment score was not a criterion for study selection but was to be considered in the determination of the robustness of our data synthesis.

### Data synthesis

The extracted data were first categorised broadly as either general population or occupational cohort studies. Thereafter, they were summarised as either observational or experimental/intervention studies. The observational studies were then further organised according to study design (cross-sectional/case–control and prospective), and experimental/intervention studies were categorised as RCTs and non-RCTs to simplify the evidence synthesis. Within the categories, the SB domain measured was organised into occupational and non-occupational SB, and the measuring instrument into device-measured and self-reported SB. Further, grouping was completed according to measured SB [full-day, leisure-time, workplace sitting, computer time, vehicle time (time spent sitting in a vehicle), and sedentary behaviours (SBs) – time spent watching television, on computer/video gaming, reading or talking on the phone], as well as the type of MSP condition outcomes. The MSP conditions included back pain (low back pain – LBP and upper back pain – UBP); neck/shoulder pain; knee osteoarthritis (pain); extremities pain (upper and lower); and other MSP conditions (included MSP conditions reported no more than three in the reviewed studies; a general MSP/discomfort or collectively measured MSP conditions; and arthritis).

Descriptive tables and narrative text provide a general overview of the studies reviewed. MSP condition outcomes (e.g., back pain, neck/shoulder pain, and knee osteoarthritis) reported in three studies or more with permissible variations in the study designs and measures were quantitatively synthesised. Otherwise, the MSP condition is presented in a narrative review.

#### Narrative review

In the case whereby meta-analysis was not feasible, individual study findings were systematically described and integrated using the best-evidence synthesis in a narrative text [53, 54]. This commonly used synthesis approach takes into account the quality and the consistency of reported findings of the studies in three levels – strong evidence (≥ 75% of the studies show consistent significant findings in the same direction of ≥ 2 high-quality studies; moderate evidence (consistent significant findings in the same direction of a high-quality and at least a low-quality studies or ≥ 2 low-quality studies; and insufficient evidence (inconsistent findings in ≥ 2 studies or just a single available study). When there were ≥ 2 studies of high quality in a category, our conclusion on the evidence of associations was based on the within- and between-relationships of the high-quality studies.

#### Quantitative synthesis

Pooled meta-analysis was performed on homogenous data for SB and MSP condition outcomes when permissible. The RevMan5 (Review Manager 5.4.1) inverse-variance approach was used to estimate the pooled effect size (in odds ratio) based on random effect due to the heterogeneity of the data [55]. When there were sufficient studies, subgroup analysis was performed based on self-reported and device-measured SB. To gain insight on how occupation type could mask the association of workplace sitting with MSP conditions, a subgroup analysis by occupation type was performed. Further, subgroup analysis was conducted for studies that reported neck, shoulder, and neck/shoulder pain, and for a subgroup that reported extremities pain. Pooled effect relationships were illustrated by forest plots, and data heterogeneity was estimated by I^2^, Tau^2^, and Cochran’s Chi-square. The robustness of our estimated pooled effect sizes was examined in a sensitivity analysis by excluding studies of low quality from the estimate; we used a funnel plot to illustrate potential publication bias.

In general, evidence synthesised by narrative review (the best-evidence synthesis) or quantitative synthesis (meta-analysis) from observational studies was regarded as either of low quality for cross-sectional/case–control studies-based evidence or high quality for prospective studies-based evidence. Evidence synthesised from experimental/intervention studies was regarded as of moderate/high quality depending on the relative contribution of non-RCT and RCT studies in the evidence.

## Results

The search identified 5060 studies (Fig. 1) and 3690 remained after removing duplicates. These studies were screened by title and abstract according to the review’s inclusion and exclusion criteria. A total of 178 studies were retained for full-text screening. Of these, we excluded 99 studies (Supplementary Table 2: Studies excluded after full-text screening) after the full-text screening, leaving 79 studies published from 2000 to 2021 for the evidence synthesis, including 26 studies for meta-analysis. The included studies had representation from 36 different countries. Several of these countries were the settings for five or more studies: Australia (10), Denmark (8), Brazil (8), South Korea (5), the USA (5), and the UK (5).

### Characteristics of the included studies

The characteristics of the studies are detailed in Tables 1, 2, and 3 for the general population cohorts, observational occupational cohorts, and experimental/intervention occupational cohorts, respectively. Overall, 24 observational studies were categorised as general population cohort studies; 55 studies as occupational cohort studies, which included 40 observational studies and 15 experimental/intervention studies. The occupational category comprised studies of office workers (21); professionals – physicians, specialists, nurses, university staff, teachers, students, and police duty officers (20); tradespeople and manual workers – construction, factory, manufacturing, cleaning, transport, handicraft, sewing machine operators, steel plant workers and beauticians (14); and bus drivers (3), included a study [56] that recruited office workers, professionals, and tradespeople; and another study [57] was also of professionals and tradespeople. Cross-sectional designs and a case–control design accounted for 75% and prospective designs 25% in the general population category, whereas 85% of the observational studies in the occupational category were cross-sectional and 15% had prospective designs. Among the experimental/intervention studies, however, there were six randomised controlled trials (RCTs), two randomised cross-over trials, and two non-randomised experiment without control; one study each of non-RCT, randomised trial (RT) without control, non-RT without control (a pilot study), non-randomised cross-over trial, and a cross-sectional analysis of a dataset from an RCT.

**Table 1:** Characteristics of the general population studies

**Study ID + Country**	**Study population + Duration + Sample size + Average age/ BMI + %Female + Study name**	**Sedentary behaviour (SB) domain + Measures**	**Musculoskeletal pain (MSP) conditions + % Prevalence + Measures**	**Statistical analysis + Adjusted covariates**	**Conclusions on associations of SB with MSP conditions + Effect size/*p*-value**	**Quality score**
**Study design – cross-sectional**						
***Non-occupational Sedentary Behaviour***						
Aweto et al. 2016 [58] Nigeria	51 – 80 years Sample size = 182 Average: age = 70.17(8.62), BMI = NR %Female: 54.95%	Non-occupational – Sedentary behaviours (TV, reading, listening to music, sitting in a car, lying, talking on the phone) Self-reported	LBP, UBP, Shoulder pain, Neck pain, Knee pain, Ankle pain, Elbow pain, Arm pain – Point and 12-month prevalence %Prevalence: point prevalence = 51.6%; 12-months prevalence = 87.4% Self-reported	Chi-square (χ2) test	Positive associations of sedentary behaviours with LBP; UBP; Knee pain; and Ankle pain. No association with Neck/shoulder and Elbow pain **LBP:** χ2 = 15.7, *p*-value = 0.02; **UBP:** χ2 = 13.6, *p*-value = 0.03; **Knee pain:** χ2 = 16.8, *p*-value = 0.01; **Ankle pain:** χ2 = 14.2, *p*-value = 0.03; **Shoulder pain:** χ2 = 10.6, *p*-value = 0.56; **Neck pain:** χ2 = 7.8, *p*-value = 0.62; **Elbow pain:** χ2 = 5.6, *p*-value = 0.72	0.41
Kang et al. 2020 [59] South Korea	≥ 50 years Sample size = 3,761 Average: age = NR, BMI = NR %Female: 48.3%	Non-occupational – Total SB(≥ 7.5 h/day) Self-reported	Orthopaedic problems (OPPs): LBP, knee pain, and hip pain – 3-month prevalence %Prevalence: men – 17.7% OPPs; women – 28.6% OPPs Self-reported	Multiple logistic regression Adjusted for age, education, income, occupation, marital status, smoking, BMI, physical activity at work, leisure physical activity, alcohol, sleep duration	Positive association of total SB (≥ 7.5 h/day) with OPPs in *men* [OR(95%CI) = 1.45(1.08 – 1.93)], and no association in *women* [OR(95%CI) = 1.04(0.80 – 1.35)] *Men* had a positive association with **knee pain** [OR(95%CI) = 1.80(1.11 – 2.92)], whereas *women* had a positive association with **hip pain** [OR(95%CI) = 2.05(1.35 – 3.11)] No associations of total SB (≥ 7.5 h/day) with LBP in both men and women, knee pain in women, and hip pain in men	0.91
Kim, 2019 [60] South Korea	≥ 65 years Sample size = 301 Average: age = 72.93(0.11), BMI = NR %Female: 58.3% Korea’s 6th National Health and Nutrition Examination Survey (KNHANES VI)	Non-occupational – Total SB (≥ 7.5 h/day) Self-reported	LBP; Osteoarthritis; Knee pain; Hip pain – 3-month prevalence %Prevalence: LBP = 30.5; Osteoarthritis = 92.7; Knee pain = 27.3; Hip pain = 12.8 Self-reported	Multiple logistic regression Adjusted for sex, age, obesity, housing type, family income, education, and marital status	Positive associations of total SB (sitting) with LBP, knee pain, hip pain; and no association with osteoarthritis **LBP:** OR(95%CI) = 1.44(1.19 – 1.74), *p* < 0.001; **Knee pain:** OR(95%CI) = 1.41(1.11 – 1.79), *p* < 0.05; **Hip pain:** OR(95%CI) = 1.54(1.1 – 2.03), *p* < 0.05; **Osteoarthritis:** OR(95%CI) = 1.72(0.86 – 3.43), *p* = 0.126	0.91
Kulaivelan et al. 2018 [61] India	All adults Sample size = 1503 Average: age = 48.23(13.12), BMI = 25.97(4.57) %Female: 54.2%	Non-occupational – TV time, TB SB (sitting) Self-reported	LBP – 12-month prevalence %Prevalence: 9.0% Self-reported – MNMQ	Binary logistic regression Adjusted for smoking, income, sleeping hours, scheduled caste	No associations of TV time and total SB (sitting) with LBP **Sitting time (upper quartile):** OR(95%CI) = 1.17(0.85 – 1.62); **TV time(> 2 h/day**): OR(95%CI) = 1.17(0.82 – 1.66)	0.68
Lee et al. 2019 [16] South Korea	≥ 50 years Sample size = 8008 (Without chronic pain = 6344, chronic pain = 1664) Average: age – without chronic knee pain = 65.2(9.3), chronic knee pain = 61.3(8.7); BMI – without chronic knee pain = 24.0(3.1), chronic knee pain = 24.7(3.3) %Female: without chronic knee pain = 72.6%, chronic knee pain = 27.4% KNHANES VI	Non-occupational – Total SB (< 5, 5–7, 8–10, and > 10 h/day) Self-reported – IPAQ	Chronic knee pain – 3-month prevalence %Prevalence: 20.8% Self-reported	Multivariable logistic regression Adjusted for age and BMI, individual factors (lifestyle factors and health factors), such as smoking, alcohol consumption, occupation, education, household income, physical activity, depression, and sleep duration	Total SB (> 10 h/day) is significantly positively correlated with chronic knee pain, especially in women even with high levels of physical activity **Total SB > 10 h/day** – *Overall:* OR(95% CI) = 1.28(1.02 – 1.61), *p* = 0.03; *Women:* OR(95% CI) = 1.33(1.02 – 1.74), *p* = 0.04; *Men:* OR(95% CI) = 1.17(0.78 – 1.75), *p* = 0.46	0.95
Loprinzi, 2014 [62] USA	≥ 65 years Sample size = 1753 Average: age – T2D = 73.4, without diabetes = 74.3; BMI – diabetes = 30.2, without diabetes = 27.3 %Female: diabetes = 55.1%, without diabetes = 74.3%, All = 57.4% National Health and Nutrition Examination Survey (NHANES)	Non-occupational – Total SB Device-measured – ActiGraph	Arthritis %Prevalence – With diabetes = 43.4%; without diabetes = 33.5% Self-reported	Wald tests and design-based likelihood ratio tests were used to examined statistical differences Adjusted for gender, age, and accelerometer wear time	Positive association of total SB with arthritis in both T2D and non-diabetes ***P*-value:** T2D = 0.001; without diabetes < .0001	0.91
Machado et al. 2018 [63] Brazil	≥ 65 years Sample size = 378 Average: age = 75.5(6.1), BMI = 27.3(4.9) %Female: 70.9% The PAINEL Study	Non-occupational – Total SB Self-reported	LBP – 12-month prevalence %Prevalence: 9.3% Self-reported	Logistic regression Adjusted for age, gender, BMI, income, multimorbidity, depressive symptoms, sleep hours, years of schooling, smoking, physical activity level	No association of total SB with LBP **Sitting time 4.2(2.5) h/day:** OR(95%CI) = 1.03(0.81 – 1.31)	0.73
Mendonça et al. 2020 [64] Brazil	All adults – Severely obese Sample size = 150 Average: age = 39.6(0.7), BMI = 46.1(0.5) %Female: 85.3% ‘DieTBra Trial’	Non-occupational – Total SB (Low SB < 1,182.15 min/day) Device-measured – ActiGraph	MSP –Neck, shoulders, elbows, upper back, lower back, wrist/hands, hips/thighs, knees, and ankles/feet %Prevalence: 89.3%(site with high prevalence – ankle/feet = 68.7%), LBP = 62.7%, knees = 53.3%, and UBP = 52.0%) Self-reported	Poisson regression Adjusted for sex, age, skin colour, years of schooling, economic class, and occupation	Low total SB (< 1,182.15 min/day) is associated with hip pain, but no association with shoulder pain and wrist/hands pain **Hip pain:** PR(95%CI) = 1.84(1.05 – 3.21), *p* = 0.032. **Shoulder pain:** PR(95%CI) = 1.76(0.96 – 3.23), *p* = 0.066; **Wrist/hands:** PR(95%CI) = 0.59(0.33 – 1.06), *p* = 0.078	0.95
Mendonça et al. 2020a [65] Brazil	All adults – Severely obese Sample size = 150 Average: age = 39.57(0.72), BMI = 46.12(0.53) %Female: 85.33% ‘DieTBra Trial’	Non-occupational – Total SB(Low SB < 1,182.15 min/day); Device-measured – ActiGraph	MSP-related pain intensity %Prevalence: pain – 89.33%, severe pain – 69.33%, and pain in four or more sites – 53.33% Self-reported	Poisson regression Adjusted for demographic data (gender, education, and economic class), diet and exercise (fruit and vegetable consumption and MVPA [min/day]), and clinical characteristics (falls in the last 12 months, fracture, anxiety, depression, arthritis/arthrosis, use of analgesics, and muscle relaxant use)	A longer duration of total SB is associated with the experience of more pain **SB < Median (1182.15): ***Pain* – PR(95%CI) = 0.95(0.86 – 1.06), *p* = 0.399; *Severe pain* – PR(95%CI) = 1.09(0.88 – 1.35), *p* = 0.432; *Four or More Painful Sites* – PR(95%CI) = 1.06(0.79 – 1.44), *p* = 0.680	0.91
Park et al. 2018 [66] South Korea	≥ 50 years Sample size = 5364 Average: age = without LBP = 63.4(8.7), LBP = 67.3(9.1); BMI = without LBP = 24.1(3.1), LBP = 24.4(3.4) %Female: without LBP = 52.3%; LBP = 74.2% KNHANES	Non-occupational – Total SB Self-reported – IPAQ	LBP – 3-month prevalence %Prevalence: 22.8% Self-reported	Multiple logistic regression Adjusted for age, sex, BMI, socioeconomic factors, education, household income, smoking, alcohol, and comorbidities	Positive association of total SB with LBP **Sitting time > 7 h/day:** OR(95%CI) = 1.33 (95% CI, 1.10 – 1.61)	0.95
Ryan et al. 2017 [67] UK	All adults Sample size = 2313 Average: age = 52(18), BMI = 28(5) %Female: 55% Health Survey for England (HSE)	Non-occupational – Total SB Device-measured – ActiGraph	Chronic MSP %Prevalence: 17% Self-reported	Isotemporal substitution Adjusted for age, sex, socioeconomic status, diet, smoking history, alcohol intake, anxiety/depression, and presence of anon-musculoskeletal long-standing illness	Replacing 30 min SB with 30 min MVPA has a small but clinically relevant protective association with the chronic MSP prevalence ratio **Substituting 30 min SB with 30 min MVPA:** PR(95%CI) = 0.71(0.55 – 0.88)	0.95
Sagat et al. 2020 [68] Saudi Arabia	18 – 64 years Sample size = 463 Average: age = NR, BMI = NR %Female: 44.1%	Non-occupational – Total SB (Sitting always or most of the time) Self-reported	LBP intensity %Prevalence: Before quarantine = 38.8%, During quarantine = 43.8% Self-reported	Spearman test for correlation	A significant positive correlation of LBP intensity with sitting during Covid-19 quarantine **Correlations of LBP intensity with sitting:** *Before quarantine* – *r* = 0.054, *p* = 0.216; *During quarantine* – *r* = 0.124, *p* = 0.008	0.59
Smuck et al. 2014 [69] USA	All adults Sample size = 6796 Average: age = NR, BMI = NR %Female: NR NHANES	Non-occupational – Total SB, sedentary bout Device-measured –ActiGraph	LBP – 3-month prevalence %Prevalence: NR Self-reported	Adjusted weighted logistic regression Adjusted for BMI	Positive association of total SB and mean sedentary bout with LBP **Maximum SB bout [1239(903) min]:** OR(95%CI) = 1.03(1.1 – 1.8); **Average SB bout [50.0(46.9) min]**: OR(95%CI) = 1.09(1.3 – 3.0)	0.91
Vancampfort et al. 2017 [70] China, Ghana, India, Mexico, Russia, and South Africa	≥ 50 years Sample size = 34,129 (China = 13,175; Ghana = 4305; India = 6560; Mexico = 2313; Russia = 3938; South Africa = 3838) Average: age = median (IQR): 62(55 –70) years, BMI = NR %Female: 52.1% SAGE	Non-occupational – Total SB (≥ 8 h per day) Self-reported	Chronic LBP – 1-month prevalence %Prevalence: 8.6%, Arthritis %Prevalence: 29.5% Self-reported	Multivariable logistic regression Adjusted for sex, age, education, wealth, setting, unemployment, living arrangement, and country, comorbid chronic conditions	Positive association of total SB with arthritis and chronic LBP *Arthritis*** Overall:** OR(95%CI) = 1.22(1.03 – 1.44); **50-64 years:** OR(95%CI) = 1.17(0.92 – 1.49); ≥ **65 years:** OR(95%CI) = 1.33(1.07 – 1.67); *Chronic LBP*** Overall:** OR(95%CI) = 1.70(1.37 – 2.11), **50-64 years:** OR(95%CI) = 1.38(0.98 – 1.95), ≥ **65 years:** OR(95%CI) = 1.87(1.43 – 2.44)	0.86
***Occupational Sedentary Behaviour***						
Anita et al. 2019 [71] Spain	Born between 1940 and 1966 (> 50 years) Sample size = 1059 Average: age = 56.7(7.1), BMI – LBP = 27.1(5.4), No LBP = 27.1(4.2) %Female: 55%	Occupational – Workplace sitting Self-reported	LBP – 1-month prevalence %prevalence = 14.2% Self-reported	Multivariate regression Adjusted for age, sex, depression/anxiety level	No association of workplace sitting with LBP OR(95%CI) = 0.28(0.05 – 1.38), *p* = 0.12	0.77
***Occupational and Non-occupational Sedentary Behaviour***						
Bento et al. 2019 [72] Brazil	All adults Sample size = 600 Average: age = NR, BMI = NR %Female: 50%	Occupational—Workplace sitting; and Non-occupational—Sedentary behaviours (time spent on TV, on a computer, and/or video games) Self-reported	LBP – Point prevalence %Prevalence: 28.8% Self-reported	Poisson regression Adjusted for age, education, ethnicity, income, smoking, physical activity, depression, hypertension, diabetes, gastrointestinal, renal, and respiratory diseases	No associations od sedentary behaviours nor workplace sitting with LBP **TV time ≥ 3 h:*** Female* PR = 0.96(95%CI = 0.31 – 1.71); *Male* PR(95%CI) = 1.06(0.68 – 1.65); **Computer/video game ≥ 3 h:*** Female* PR(95%CI) = 0.70(0.37 – 1.31); *Male* PR(95%CI) = 0.52(0.24 – 1.14). **Sitting position at work (Always/usually):*** Female* PR(95%CI) = 1.24(0.90 – 1.72); *Male* PR(95%CI) = 0.88(0.56 – 1.38)	0.86
Dos Santos et al. 2017 [73] Brazil	All adults Sample size = 600 Average: age = NR, BMI = NR %Female: 50%	Occupational – Workplace sitting; and Non-occupational – sedentary behaviours (time spent on TV, on a computer, and/or playing video games) Self-reported	Neck pain – 12-month prevalence %prevalence: 20.3% Self-reported – NMQ	Poisson regression to calculate prevalence ratio with a confidence interval Adjusted for gender	No associations of workplace sitting, TV time, and computer time with neck pain **Sitting position (Always/usually):** PR = 1.09(95%CI = 0.78 – 1.52); **TV time > 3 h:** PR = 0.89(95%CI = 0.64 – 1.23); **Computer time > 3 h:** PR = 1.20(95%CI = 0.71 – 2.02)	0.77
**Study design – case–control**						
***Occupational Sedentary Behaviour***						
Pope et al. 2003 [74] UK	All adults Sample size = 3385 Average: age = NR, BMI = NR, %Female: Cases = 63.6; Control = 49.4	Occupational – Workplace sitting (≥ 2 h without a break) Self-reported	Hip pain – 1-month prevalence %Prevalence: 10.5% Self-reported	Logistic regression Adjusted for age, sex, and all physical activities	Positive association of prolonged sitting with hip pain **Sitting for prolonged periods – ≥ 2 h:*** (higher exposure vs not exposed):* OR(95%CI) = 1.82(1.13 – 2.92)	0.91
**Study design – prospective**						
***Non-occupational Sedentary Behaviour***						
Balling et al. 2019 [75] Denmark	All adults Duration: mean 7.4-years Sample size = 46,826 Average: age = 47.6(15.8), BMI = 24.8(4.2) %Female: 60.3%	Non-occupational – Total SB (sitting time) Self-reported – IPAQ	LBP – Incidence %Incidence: 3.8% Medical records	Cox regression Adjusted for age, sex, mental disorder, education, smoking status, BMI, leisure-time physical activity, and physical activity at work	No association of total SB (sitting) with an incidence of LBP **Sitting 6 to < 10 h:** HR(95%CI) = 0.99(0.89 – 1.10); **10 + hrs:** HR(95%CI) = 0.99(0.86 – 1.16)	0.95
Chang et al. 2020 [76] USA	45 – 79-years at baseline Duration: 8-year Sample size = 1194 Average: age = 58.4(8.9), BMI = 26.8(4.5) %Female: 58.4% Osteoarthritis Initiative (OAI)	Non-occupational – Extensive sitting behaviour over 8 years Self-reported	Knee pain – 12-month incidence %Incidence: 13.0% Clinical diagnosis – radiologic examination	Logistic regression Adjusted for age, gender, BMI, depressive symptoms, comorbidities	No association of extensive sitting trajectory with incident knee osteoarthritis **Moderate frequency sitting trajectory:** RR(95%CI) = 1.02(0.88 – 1.18); **High frequency sitting trajectory:** RR(95%CI) = 1.22(1.00 – 1.50)	0.95
da Silva et al. 2019 [77] Australia	All adults Duration: 3-, 6-, 9- and 12-month follow-ups Sample size = 250 Average: age = 50(15), BMI: 26.5(5.3) %Female: 50%	Non-occupational – Total SB Self-reported	LBP – Incidence %Incidence: 38% at 3-months; 56% at 6-months; and 69% at 12-months Self-reported – 11-point numerical rating scale	Cox regression – completeness of follow-up was calculated using the completeness index Adjusted for age BMI, smoking, and exposure to heavy load	Positive association of sitting time with LBP **Sitting > 5 h:** HR(95%CI) = 1.50(1.08 – 2.09), p = 0.02	0.73
Hussain et al. 2016 [78] Australia	All adults Duration: 5-, 12-years Sample size = 4974 Average: age = NR, BMI = NR %Female: 55.8% Australian Diabetes, Obesity, and Lifestyle (AusDiab) Study	Non-occupational – TV time Self-reported	LBP intensity, LBP disability – 6-month prevalence %Prevalence: 81.9% Self-reported – Chronic Pain Grade Questionnaire (CPGQ)	Multinomial logistic regression Adjusted for age, education, smoking status, dietary guideline index score, and BMI; SF-36 MCS score	High levels of TV time are positively associated with an increased risk of LBP disability in women but not in men. No association of TV time with LBP intensity **TV time ≥ 2 h: *LBP intensity**** (Men)* Low: OR(95%CI) = 1.15(0.91 – 1.46), *p* = 0.25; High: OR(95%CI) = 1.17(0.86 – 1.59), *p* = 0.31; *(Women)* Low: OR(95%CI) = 1.11(0.88 – 1.40), *p* = 0.37; High: OR(95%CI) = 1.17(0.88 – 1.56), *p* = 0.28; ***LBP Disability**** (Men)* Low: OR(95%CI) = 1.10(0.84 – 1.43), *p* = 0.50; High: OR(95%CI) = 1.15(0.82 – 1.61), *p* = 0.42; *(Women)* Low: OR(95%CI) = 1.35(1.04 – 1.73), *p* = 0.02; High: OR(95%CI) = 1.29(1.01 – 1.72), *p* = 0.04	0.82
Stefansdottir & Gudmundsdottir, 2017 [17] Iceland	All adults Duration: 5-years Sample size = 737 Average: age = 53(16), BMI = 27(5) %Female: 39% Health and Wellbeing of Icelanders survey	Non-occupational – Total SB Self-reported	General musculoskeletal symptoms – 5-year prevalence %Prevalence: 33.5% Self-reported	Not reported	Positive association of total SB with general MSP **High SB:** OR(95%CI) = 1.7(1.03 – 2.83)	0.50
***Occupational Sedentary Behaviour***						
Martin et al. 2013 [79]UK	36-year, 43-year, and 53-year old cohorts Duration: Since birth in 1946 Sample size = 2957 Average BMI: 36-year = 24.1(3.7), 43-year = 25.4(4.2), 53-year = 27.4(4.8) %Female: 36-year = 51.3%, 43-year = 51.3%; 53-year = 50.7%	Occupational – Workplace sitting (> 2 h) Self-reported	Knee pain (Osteoarthritis) – 1-month prevalence %Prevalence: 10.2% Self-report and clinical examination	Logistic regression Adjusted for gender, health risk factors, and socioeconomic position	Negative association of workplace with knee osteoarthritis in women, but no association in men **Sitting highly likely:*** (Men) 36 years* OR(95%CI) = 1.13(0.61 – 2.06), *p* = 0.700; *43 years* OR(95%CI) = 0.69 (0.39 – 1.24), *p* = 0.226; *53 years* OR(95%CI) = 0.60 (0.34 – 1.07), *p* = 0.085; *(Women) 36 years* OR(95%CI) = 0.56 (0.33 – 0.94), *p* = 0.029; *43 years* OR(95%CI) = 0.57 (0.36 – 0.89), *P* = 0.013; *53 years* OR(95%CI) = 0.89 (0.56 – 1.43), *p* = 0.653	0.91

NR: Not reported, (M)NMQ: (Modified) Nordic musculoskeletal questionnaire, TV: Television-viewing

**Table 2:** Characteristics of the observational occupational cohort studies

**Study ID + Country**	**Study population + Duration + Sample size + Average age/ BMI + %Female + Study name**	**Nature of occupation**	**Sedentary behaviour (SB) domain + measures**	**Musculoskeletal pain (MSP) conditions + % Prevalence + Measures**	**Statistical analysis + Adjusted covariates**	**Conclusions on associations of SB with MSP conditions + Effect Size/*p*-value**	**Quality score**
**Study design – cross-sectional**							
***Occupational Sedentary Behaviour***							
Ayanniyi et al. 2010 [80] Nigeria	All adults Sample size = Computer users = 236; Non-computer users = 236; Total = 472 Average: age – Computer users = 29(4.87), Non-computer users = 31(6.23); BMI = NR %Female: Computer users = 42.4%; Non-computer users = 42.4%	Office workers	Occupational – Computer time Self-reported	Musculoskeletal symptoms (Neck/shoulder pain, UBP, elbows, wrists/hands, LBP, hips/thighs, knees, and ankles/feet pain) – 7- and 12-month prevalence %Prevalence: 7 days point prevalence – Computer users = 55.9%, Non-computer users = 27.5%; 12-months prevalence – Computer users = 93.2%, Non-computer users = 33.9% Self-reported	Regression analysis Adjusted for age, sex, marital status	Positive association of computer time with musculoskeletal symptoms **7-day prevalence:*** 2–4 h* – OR = 1.36(95%CI = 0.92 – 1.68, *p* < 0.05); > *4 h* – OR = 4.12(95%CI = 3.21 – 5.16, *p* < 0.05); **12-Month prevalence:*** 2–4 h* – OR = 3.25(95%CI = 1.84 – 4.73, *p* < 0.05); > *4 h* – OR = 5.04(95%CI = 3.66 – 6.33, *p* < 0.05)	0.73
Benyamina et al. 2018 [81] Canada	All adults Sample size = 2208 Average: age = 35.8(8.1), BMI = NR %Female: 31.1%	Professionals – Car-patrol police officers	Occupational –vehicle time (time spent sitting in a vehicle) Self-reported	LBP – 12-month prevalence %Prevalence: Chronic LBP = 28.1%, acute/subacute LBP = 40.7% Self-reported – NMQ	Multinomial regression Adjusted age, sex, country of birth, income, the region of residency, depressed mood, and anxiety	No association of vehicle time with LBP **Acute/subacute LBP vs No-LBP:** OR(95%CI) = 1.005 (0.998 – 1.012), *p* = 0.169; **Chronic LBP vs No-LBP**: OR(95%CI) = 1.002 (0.993 – 1.010), *p* = 0.702	0.77
Cagnie et al. 2007 [82] Belgium	All adults Sample size = 512 Average: age = NR, BMI = 24.0(3.4) %Female: 41.7%	Office workers	Occupational – prolonged workplace sitting and computer time (> 4 h/day) Self-reported	Neck pain – 12-month prevalence %Prevalence: 45.5% Self-reported – NMQ	Logistic regression Adjusted for age, gender, mental tiredness, and sport	Positive associations of prolonged workplace sitting and computer time with neck pain **Workplace sitting:** OR(95% CI) = 2.06(1.17 – 3.62); **Computer time:** OR(95% CI) = 1.57(1.10 – 2.22)	0.73
Celik et al. 2018 [83] Turkey	All adults Sample size = 528 Average: age = 38.55(9.79), BMI = 25.44(3.85) %Female: 51.14%	Office workers	Occupational – Total workplace sitting [mean = 4.64(2.21) Self-reported	LBP, UBP, Shoulder pain, Neck pain, Leg pain, Arm pain, Foot pain, Wrist pain %Prevalence: LBP – Female = 60.4%, Male = 49.6%; UBP – Female = 62.6%, Male = 43.0%; Shoulder pain – Female = 50.0%, Male = 31.0%; Neck pain – Female = 61.9%, Male = 42.6%; Leg pain – Female = 39.6%, Male = 26.7%, Arm pain – Female = 33.0%, Male = 20.5%; Foot pain – Female = 45.6, Male = 37.2%; Wrist pain – Female = 33.7%, Male = 19.0% Self-reported	Multiple-linear regression Adjusted for age, BMI, marital status, exercise in daily life, working experience	No significant association of workplace sitting with LBP, UBP, shoulder and wrist pain. Negative association of workplace sitting with neck and extremities pain (arm, leg, and foot) in females **LBP:*** Female* B = –0.07, SE = 0.04, 95%CI = –0.16–0.00, *p* = 0.080; *Male* B = –0.03, SE = 0.04, 95%CI = –0.13–0.05, *p* = 0.458; **UBP:*** Female* B = –0.00, SE = 0.04, 95%CI = –0.09–0.07, *p* = 0.825; *Male* B = 0.06, SE = 0.04, 95%CI = –0.03–0.15, *p* = 0.195; **Neck pain:*** Female* B = –0.110, SE = 0.04, 95%CI = –0.20–(–0.02), *p* = 0.009; *Male* B = 0.04, SE = 0.04, 95%CI = –0.04–0.13, *p* = 0.352. **Shoulder pain:*** Female* B = –0.02, SE = 0.04, 95%CI = –0.10–0.06, *p* = 0.648;* Male* B = 0.01, SE = 0.04, 95%CI = –0.06–0.10, *p* = 0.711; **Leg pain:*** Female* B = –0.08, SE = 0.04, 95%CI = –0.170–0.000, *p* = 0.043; *Male* B = –0.01, SE = 0.04, 95%CI = –0.11–0.07, *p* = 0.687; **Foot pain:*** Female* B = –0.09, SE = 0.040, 95%CI = –0.18–(–0.01), *p* = 0.027; *Male* B = 0.00, SE = 0.04, 95%CI = –0.07–0.08, *p* = 0.891; **Arm pain:*** Female* B = –0.10, SE = 0.04, 95%CI = –0.18–(–0.02), *P* = 0.010; *Male* B = 0.00, SE = 0.04, 95%CI = –0.07–0.08, *p* = 0.919; **Wrist pain:*** Female* B = –0.04, SE = 0.04, 95%CI = –0.12–0.04, *p* = 0.343; *Male* B = 0.03, SE = 0.03, 95%CI = –0.03–0.11, *P* = 0.292	0.73
Chee & Rampal 2004 [84] Malaysia	All adults Sample size = 906 Average: age = NR, BMI = NR %Female: 100%	Tradespeople – Semiconductor factory workers	Occupational – Workplace sitting (≥ 4 h/day) Self-reported	Neck/shoulder pain, and lower limbs – 12-month prevalence %Prevalence: 80.5% Self-reported – NMQ	Multivariate binary logistic regression Adjusted for age, work task, work schedule, overtime work, whether work environment was too cold, and stress	Positive association of workplace sitting with **neck/shoulder pain** [OR(95% CI) = 1.6(1.2 – 2.1)]; a negative association with **Lower limbs** OR(95% CI) = 0.5(0.4 – 0.8)	0.91
Chrasakaran et al. 2003 [85] Malaysia	All adults Sample size = 529 Average: age = 31.2(7.4), BMI = NR %Female: 100%	Tradespeople –Semiconductor factory workers	Occupational – Workplace sitting (≥ 4 h/day) Self-reported	Neck, shoulder, arm (elbow and forearm), wrist and fingers, upper leg (hips/thighs/knees), lower leg (ankles/feet) – 12-month prevalence %Prevalence: lower leg (48.4%), shoulder (44.8%), upper leg (38.8%) and neck (29.7%) Self-reported – NMQ	Logistic regression Adjusted for age, number years of work, the stress of work, cold working temperature	Positive association of workplace sitting with NSP, but no association with extremities pain **Neck:** [OR(95% CI) = 2.1(1.3 – 3.2); **Shoulder:** [OR(95% CI) = 1.7(1.2 – 2.5); **Upper leg:** [OR(95% CI) = 0.6(0.3 – 1.0); **Lower leg:** [OR(95% CI) = 0.6(0.4 – 1.0)	0.73
Constantino et al. 2019 [86] Brazil	All adults Sample size = 530 Average: age = NR, BMI = NR %Female: 95.4%	Professionals – Teachers	Occupational – workplace sitting (≥ 2 h/day), computer time(≥ 2 h/day); and Non-occupational – TV time(≥ 2 h/day) Self-reported	Clinically diagnosed MSP disease; musculoskeletal symptoms (back/neck); and MSP-related disability – 12-month prevalence %Prevalence: > 30% Self-reported – NMQ	Poisson regression Adjusted for age, gender, length of employment, high stress, common mental disorder, physical activity	Negative association of workplace sitting with **lower limbs disability** [Adjusted PR(95%CI) = 0.64(0.43–0.94)]; No association of TV time with **back & neck pain** [Adjusted PR(95%CI) = 1.03(0.88–1.21)]; Positive association of TV time with clinically diagnosed **MSP disease** [Adjusted PR(95%CI) = 1.37(1.02–1.85)]; No association of computer time with clinically diagnosed **MSP disease** [Adjusted PR(95%CI) = 0.78(0.60–1.02)]	0.77
Dianat & Karimi, 2016 [87] Iran	All adults Sample size = 632 Average: age = 34.5(11.5), BMI = 24.9(4.1) %Female: 58.9%	Tradespeople – Handicraft workers	Occupational – Workplace sitting (> 2 h/day) Self-reported	Neck, shoulders, LBP –1-month prevalence %Prevalence: 76.2% Self-reported – NMQ	Logistic regression Adjusted for age, gender, BMI, marital status, education level, smoking, physical activity, years working	Positive association of workplace sitting > 2 h with **neck pain** in multivariate analysis [OR(95% CI) = 2.85(1.79 – 4.53), *p* < 0.001] Univariate analysis showed a positive association of workplace sitting with **shoulder pain** [OR(95% CI) = 1.54(1.02 – 2.31)];and no association with **LBP** [OR(95% CI) = 0.99(0.66 – 1.47)]	0.86
Dianat et al. 2015 [88] Iran	All adults Sample size = 251 Average: age = 33.2(9.9), BMI: 24.1(4.1) %Female: 39.8%	Tradespeople – Sewing machine operators	Occupational – workplace sitting (> 2 h/day) Self-reported	Neck, shoulders, UBP, LBP, elbows, wrists/hands, hips/thighs/buttocks, knees, and ankles/feet – 12-month prevalence %Prevalence: 9.6% Self-reported – NMQ	Logistic regression Adjusted for demographic (age, gender, BMI, educational level, marital status, smoking, physical activities, and job characteristics, and RULA scores	Positive association of workplace sitting > 2 h with **neck pain** [OR(95% CI) = 3.34(1.40 – 7.95), *p* = 0.006]; and s**houlder** **pain** [OR(95% CI) = 3.12(1.19 – 8.18), *p* = 0.020] in multivariate analysis However, univariate analysis showed no association of workplace sitting with **LBP** [OR(95% CI) = 1.12(0.41 – 2.99), *p* = 0.821], and **UBP** [OR(95% CI) = 1.04(0.93 – 1.16), *p* = 0.102]; but positive association with **Hand/wrist** [OR(95% CI) = 2.49(1.08 – 5.72), *p* = 0.031]	0.86
Ilic et al. 2021 [89] Serbia	Young to middle-aged Sample size = 499 Average: age = 22.0(2.2) %Female: 67.7%	Professionals – Students	Occupational – Workplace sitting (prolonged sitting) Self-reported	LBP – Point prevalence %Prevalence: 20.8% Self-reported	Logistic regression Adjusted for smoking, BMI, Incorrect body posture, stress, incorrect sitting position, family history of LBP	Multivariate analysis: No association of prolonged sitting with LBP [OR (95%CI) = 1.5(0.5 – 4.2), *p* = 0.424 Univariate analysis – prolonged sitting associated with LBP (*p* = 0.018)	0.82
Hakim et al. 2017 [90] Egypt	All adults Sample size = 180 Average: age = NR, BMI = NR %Female: 0%	Bus divers	Occupational – vehicle time (> 8 h/day) Self-reported	LBP – 12-month prevalence %Prevalence: 73.9% Self-reported – NMQ	Binary logistics regression Adjusted for age, BMI, marital status, education, smoking, work duration	Positive association of vehicle time (> 8 h) with LBP OR(95%CI) = 2.93(1.45 – 5.93)	0.68
Larsen et al. 2018 [91] Sweden	All adults Sample size = 4114 Average: age = NR, BMI = NR %Female: 25.8%	Professionals – Duty police officer	Occupational – Vehicle time (% shift time sitting: 25 – 50%, 50 – 75%, > 75%) Self-reported	Multisite MSP (pain in two or more body regions) – 3-month prevalence %Prevalence: 41.3% Self-reported – 5-point scale	Binominal logistic regression.; adjusted for age, sex, physical exercise, physical workload factors, and psychosocial factors	Vehicle time vehicles were not significantly associated with multi-site MSP among police **Shift time sitting:*** 25* – *50%* OR(95%CI) = 0.97(0.74 – 1.28); *50 – 75%* OR(95%CI) = 1.11(0.84 – 1.47); > *75%* OR(95%CI) = 1.10(0.77 – 1.57)	0.86
Lourenço et al. 2015 [92] Portugal	21-year cohorts Sample size = 1733 (Non-workers = 1083; Workers = 650) Average BMI = NR %Female: Non-workers = 51.8%; Workers = 51.2% Epidemiological Health Investigation of Teenagers in Porto (EPI-Teen)	Professionals – Student	Occupational – Workplace sitting (> 4.2 h/week); computer time (> 5.0 h/week) Self-reported	Neck, shoulders, elbows, wrists/hands, upper back, lower back, hips/thighs/buttocks, knees, and ankles/feet – 12-month prevalence Self-reported	Logistic regression Adjusted for sex, BMI, physical activity, smoking, education, and job strain (Karasek’s Job Strain Model)	A positive association of workplace sitting with **LBP** [OR(95%CI) = 1.70(1.20 – 2.42)]; no association with **neck pain** [OR(95%CI) = 1.23(0.89 – 1.71)] and **extremities pain** [OR(95%CI) = 0.83(0.60 – 1.16)]	0.91
Mehrdad et al. 2012 [93] Iran	All adults Sample size = 405 Average: age = 44.6 (7.9), BMI: 23.7(2) %Female: 47%	Professionals – physicians	Occupational – Prolonged workplace sitting (> 20 min) Self-reported	Neck pain^a^ – 12-month prevalence %Prevalence: 41.7% Self-reported – NMQ	Logistic regression Adjusted for both individual and work-related factors such as age, gender, BMI, shift work, type of employment, and secondary job	A positive association of prolonged workplace sitting with neck pain Coefficient(B) = 0.204, OR(95%CI) = 1.227(1.032 – 1.458), *p* = 0.020	0.86
Omokhodion et al. 2003 [94] Nigeria	All adults Sample size = 840 Average: age = NR, BMI = NR %Female: 43%	Office workers	Occupational – Workplace sitting (> 3 h) Self-reported	LBP – 12-month prevalence %Prevalence: 37.5%; Self-reported	Not reported	Workplace sitting for > 3 h associated with increased severity of LBP	0.36
Pradeepkumar et al. 2020 [95] India	24 – 55 years Sample size = 301 Average: age = 39(7.3), BMI = NR %Female: NR	Bus drivers	Occupational – Vehicle time (Prolonged sitting) Self-reported	MSP conditions – 7-day and 12-month prevalence %Prevalence: 55.8%; Self-reported – NMQ	Chi-square test	Prolonged sitting in a vehicle is positively associated with the risk of MSP conditions χ2 = 5.833, *p* < 0.05	0.55
Ratzon et al. 2000 [96] Israel	All adults Sample size = 60 Average: age = 46.0 (8.66), BMI – Sitting position = 25.14(2.18), Alternating position = 25.31(2.44) %Female: 0%	Professionals – Dentist	Occupational – Workplace sitting (≥ 80% of work time) Self-reported	General MSP, LBP – 7-days and 12-month prevalence %Prevalence; Low back pain = 55% Self-reported – NMQ	Pearson and Spearman correlations	Sitting position at work positively and significantly correlated with LBP Correlation coefficient – **MSP** = − 0.16; **LBP:*** r* = 0.41, *p* < 0.01	0.45
Şimşek et al. 2017 [97] Turkey	All adults Sample size = 1682 Average: age = 37.9(7.46), BMI: NR %Female: 60%	Professionals – Healthcare workers	Occupational – Workplace sitting (> 4 h), computer time (> 4 h) Self-reported	LBP – 7-days, 12-month, and lifetime prevalence %Prevalence: Lifetime prevalence 53%, 12-month prevalence 39% and 7-days prevalence 29.5% Self-reported – NMQ (10-cm-long Visual Analogue Scale (VAS))	Binary logistic regression Adjusted for sex, BMI, marital status, smoking habit, physical exercise, job satisfaction, workplace stress	Positive associations of workplace sitting and computer time > 4 h with LBP **Workplace sitting time:** OR(95%CI) = 4.7(1.25 – 17.64), *p* = 0.021; **Computer time:** OROR(95%CI) = 0.0(0.00 – 0.04), *p* = 0.0001	0.86
Spyropoulos et al. 2007 [98] Greece	All adults Sample size = 648 Average: age = 44.5, BMI = NR %Female: 75.8%	Office workers	Occupational – Workplace sitting (≥ 6 h) Self-reported	LBP – Lifetime prevalence %Prevalence: Lifetime 61.6% Self-reported – Visual Analogue Scale (VAS) and physical examination by a physiotherapist	Multiple logistic regression Adjusted for age gender, BMI, psychosocial factors	Positive association of workplace sitting time > 6 h with lifetime LBP OR(95z5CI) = 1.588(1.064 – 2.368)	0.82
Szeto & Lam, 2007 [99] Hong Kong	All adults Sample size = 481 Average: age = NR, BMI – Male = 25.24(3.42); Female = 23.60(2.74) %Female: 16%	Bus drivers	Occupational – Vehicle time (prolonged sitting) Self-reported	LBP^a^ – 12-month prevalence %Prevalence: 92.7% Self-reported – NMQ	Logistic regression Adjusted for age, gender, company	Positive association of prolonged vehicle time with LBP OR(95% CI) = 3.71(2.40 – 5.74)	0.77
Temesgen et al. 2019 [100] Ethiopia	All adults Sample size = 754 Average: age = 42(9.73), BMI = NR %Female: 57.8%	Professionals – Teachers	Occupational – Workplace sitting (prolonged sitting > 4 h/day) Self-reported	Neck/shoulder pain – 12-month prevalence %Prevalence: 57.3% Self-reported – NMQ	Logistics regression Adjusted for age, marital status, salary, smoking, alcohol, physical exercise, diabetes, hypertension, respiratory diseases	Positive association of prolonged workplace sitting > 4 h with neck/shoulder pain OR(95%CI) = 1.50(1.02 – 2.23)	0.95
Tsigonia et al. 2009 [101] Greece	All adults Sample size = 102 Average: age = 38.42(10.74), BMI = 23.09(2.86) %Female: 93%	Tradespeople – Cosmetologists	Occupational – Workplace sitting (High exposure to prolonged sitting – often or always) Self-reported	Neck, shoulder, hand/wrist, low back, knee; 12-month prevalence; %Prevalence: Neck = 58%; shoulder = 35%; hand/wrist = 53%; low back = 53%; knee = 28%; Self-reported– NMQ	Logistics regression; adjusted for age and sex	Positive association of high exposure to prolonged workplace sitting with hand/wrist complaints, OR(95%CI) = 55.7(18.75- 354.93) Univariate analysis indicates workplace sitting is significantly related to the occurrence of LBP, neck/shoulder pain, hand and knee pain (both acute and chronic complaints)	0.73
van Vuuren et al. 2005 [102] South Africa	All adults Sample size = 366 Average: age = 31.76(7.80), BMI = NR %Female: NR	Tradespeople – Steel plant workers	Occupational – Workplace sitting (sitting position half the time or more) Self-reported	LBP, LBP disability – Point, 1-month, 12-month, and lifetime prevalence %Prevalence: Point 35.8%, 1-month 41.3%, 12-month 55.7%, and lifetime 63.9%; LBP disability – ≥ 30% Self-reported – Functional Rating Index (FRI)	Multivariate logistic regression Adjusted for all risk factors including work organization, trunk posture, handling activities, body position, and environmental demands	Positive association of workplace sitting with LBP, but no significant association with LBP disability **LBP:** [OR(95%CI) = 2.33(1.01 – 5.37)]; **LBP disability:** [OR(95%CI) 1.89(0.75 – 4.78)]	0.77
Yue et al. 2012 [103] China	All adults Sample size = 893 Average: age = 32.21(10.6), BMI = 39(2.79) %Female: 67%	Professionals – Teachers	Occupational – Workplace sitting (≥ 4 h/day); Computer time (≥ 4 h/day) Self-reported	LBP, neck/shoulder pain – 12-month prevalence %Prevalence: LBP = 45.6%, NSP = 48.7% Self-reported – NMQ	Binary logistic regression Adjusted for age, gender, BMI, education, smoking, exercise, years of work, duration of work	Positive association of prolonged *workplace sitting (≥ 4 h)* with **neck/shoulder pain** [OR(95%CI) = 1.76(1.23 – 2.52)] and **LBP**[OR(95%CI) = 1.42 (1.01 – 2.02)] No significant association of *computer time (≥ 4 h)* with **neck/shoulder pain** [OR(95%CI) = 1.02 (0.63 – 1.65)] and **LBP** [OR(95%CI) = 0.71 (0.44 – 1.14)]	0.86
***Non-occupational Sedentary Behaviour***							
Ben-Ami et al. 2018 [104] Israel	All adults Sample size = 1026 Average: age = 27.2(6.4), BMI = NR %Female: 57.7%	Professionals – Students	Non-occupational – Leisure-time SB (at least half an hour a day) Self-reported	LBP – 6-month prevalence %Prevalence: 38.6% Self-reported	Multinominal logistic regression Adjusted for sociodemographic, lifestyle, and personal vulnerability	No significant association of total SB with LBP (backache) AOR(95%CI) = 0.96(0.78 – 1.18)	0.86
Hildebrandt et al. 2000 [56] Netherlands	All adults Sample size = 2030 Average: age = 33.7(9.6), BMI: NR %Female: 51%	Tradespeople – Industry (shipyard, metal, transport) and services (cleaners, childcare); Professionals – Healthcare(nurses); and Office workers	Non-occupational – Leisure-time SB Self-reported	LBP, neck/shoulder pain, and lower extremity pain – 12-prevalence %Prevalence: LBP = 60%, NSP = 44%, and lower extremity pain = 31% Self-reported	Logistic regression Adjusted for age, gender, education, and type of workload	Leisure-time SB is positively associated with **LBP** [OR(95%CI) = 1.46(1.18 – 1.29)]; and no associated with **neck/shoulder pain** [OR(95%CI) = 1.02(0.82 – 1.27)], and **lower extremities pain** [OR(95%CI) = 1.07(0.85 – 1.36)]	0.73
Ibeachu et al. 2019 [105] UK	18 – 39 years Sample size = 314 Average: age = 22.0(5.2), BMI = 24.3(4.1) %Female: 43.9%	Professionals – Student	Non-occupational – Total SB (mean 5.6(2.6)hrs/day) Self-reported – IPAQ	Knee pain – 12-month prevalence %Prevalence: 31.8% Self-reported – Knee Pain Screening Tool (KNEST)	Logistic regression Adjusted for age, gender, BMI, mental distress	Total SB has a borderline non-significant association with knee pain (*p* = 0.069) Quadratic term: OR(95%CI) = 1.02(1.00 – 1.05) Linear term: OR(95%CI) = 1.04 (0.93 – 1.16)	0.82
Rodríguez-Nogueira et al. 2021 [106] Spain	All adults Sample size = 472 Average: age – Male = 48.1(10.9); Female = 45.3(11.2) %Female: 60%	Professionals – University staff	Non-occupational – Daily sitting time (Mean daily sitting time (hrs): Male = 7(2.5); Female = 6.9(2.3)) Self-reported	General MSP – 12-month prevalence Self-reported – NMQ	Logistic regression Adjusted for age, sex, anxiety, physical activity, self-perceived stress	No significant association of daily sitting with general MSP OR(95%CI) = 0.934(0.86 – 1.01), *p* = 0.09	0.86
Sklempe et al. 2019 [107] Croatia	Young adults Sample size = 517 Average: age – 20(2), BMI = 22.3(4.3) %Female: 63.8%	Professionals – Student	Non-occupational – Total SB (mean 5(3.5)hrs/day) Self-reported – IPAQ	Musculoskeletal symptoms (neck, shoulder, upper back, and lower back) – 12-month prevalence %Prevalence: 81% Self-reported – NMQ	Point-biserial correlation coefficient	No significant association between the time spent sitting and MSP score	0.73
Tavares et al. 2019 [108] Brazil	Young to middle-aged adults Sample size = 629 Average: age – median(IQR) = LBP = 22.5(21.0 – 24.0); no LBP = 23.0(21.0 – 25.0); Average BMI = NR %Female: 72.8%	Professionals – Student	Non-occupational – Total SB Self-reported	LBP – Lifetime prevalence; %Prevalence: 81.7%; Self-reported	Chi-squared test	No association of total SB with LBP	0.59
***Occupational and Non-occupational Sedentary Behaviour***							
Gupta et al. 2015 [109] Denmark	All adults Sample size = 201 Average: age = 44.7(9.7), BMI = 26.4 (5.0) %Female: 41.8	Tradespeople – Construction workers, cleaners, garbage collectors, manufacturing workers, assembly workers, mobile plant operators, and workers in the health service sector	Occupational – Total workplace sitting (low: ≤ 2.0 h, moderate: 2.1 – 3.7 h, high: > 3.7 h); and non-occupational – Total full day sitting (low: ≤ 6.4 h, moderate: 6.5 – 8.3 h and high: > 8.3 h); Total leisure-time sitting (Low: < 4.4 h, moderate: 4.0 – 5.4 h, high: > 5.4 h Device-measured – ActiGraph	LBP intensity – 1-month prevalence Low intensity: ≤ 5 pain score; high intensity: > 5 pain score Self-reported – NMQ	Binary logistic regression Adjusted for age, gender, BMI, and smoking, job seniority, influence at work, and occupational lifting/carrying time at work	Positive associations of the total full day sitting time and leisure-time with LBP intensity, and marginally significant association of total workplace sitting with LBP intensity **Total full day sitting:** OR = 1.43(1.15 – 1.77), *p* = 0.01; **Workplace sitting:** OR = 1.34(0.99 – 1.82), *p* = 0.06; Leisure **sitting:** OR = 1.45(1.10 – 1.91), *p* = 0.01. **High total full day sitting:** OR = 3.31(1.18 – 9.28), *p* = 0.03; **High Workplace sitting:** OR = 3.26(0.89 – 11.98), *p* = 0.08; **High Leisure sitting:** OR = 5.31(1.57 – 17.90), p = 0.01	0.95
Hallman et al. 2015 [110] Denmark	All adults Sample size = 202 Average: age = NR, BMI = NR %Female: 41.8% Danish PHysical ACTivity cohort with Objective measurements (DPHACTO)	Tradespeople – Construction workers, cleaners, garbage collectors, manufacturing workers, assembly workers, mobile plant operators, and workers in the health service sector	Occupational – Mean total workplace sitting = 3.0(1.4); and Non-occupational – mean total full day sitting = 7.3 (2.1), mean total leisure-time sitting = 4.8(1.7) Device-measured – ActiGraph	Neck/shoulder pain-intensity – 1-month prevalence %Prevalence: 75.2% Self-reported – NMQ (numeric rating scale (NRS))	Logistic regression Adjusting for age and gender, individual factors (i.e., BMI and smoking), work-related factors (i.e., seniority, influence at work, and lifting/carrying)	Positive associations of the total full day sitting and workplace sitting with neck/shoulder pain intensity. Low total workplace sitting is associated with reduced neck/shoulder pain intensity in men. No association of leisure-time sitting with neck/shoulder pain intensity **Total full day Sitting:*** High sitting* (*Overall*) OR(95%CI) = 2.97(1.25 – 7.03), *p* = 0.01; *(Male)* OR(95%CI) = 6.44(1.76 – 23.56), *p* = 0.005; *(Female)* OR(95%CI) = 1.19(0.31 – 4.51), *p* = 0.44. **Workplace sitting:*** High sitting (Overall)* OR(95%CI) = 0.92(0.41 – 2.06), *p* = 0.83; *(Male)* OR(95%CI) = 0.94(0.31 – 2.85), *p* = 0.92; *(Female)* OR(95%CI) = 1.17(0.32 – 4.33), *p* = 0.82; *Low sitting (Overall)* OR(95%CI) = 0.54(0.23 – 1.25), *p* = 0.15; *(Male)* OR(95%CI) = 0.26(0.07 – 0.96), *p* = 0.04; *(Female)* OR(95%CI) = 1.01(0.28 – 3.59), *p* = 0.99. **Leisure-time:*** High sitting (Overall)* OR(95%CI) = 1.60(0.68 – 3.74) *p* = 0.28; *(Male)* OR(95%CI) = 2.76(0.83 – 9.18), *p* = 0.097; *(Female)* OR(95%CI) = 1.02(0.28 – 3.74), *p* = 0.97	0.91
Hallman et al. 2016 [111] Denmark	All adults Sample size = 659 Average: age = 45.0(9.9), BMI = 27.5(4.9) %Female: 44.9% DPHACTO	Tradespeople – Cleaning, manufacturing, transport	Occupational –workplace sitting pattern and absolute sitting time (brief: < 5 min, moderate: > 5 – 20 min, prolonged: > 20 min) and Non-occupational –leisure-time sitting pattern and absolute sitting time (brief: < 5 min, moderate: > 5 – 20 min, prolonged: > 20 min) Device-measured – ActiGraph	Neck/shoulder pain-intensity – 3-month prevalence %Prevalence: 74% Self-reported – NMQ [numeric rating scale (NRS)]	Binary logistic regression Adjusted for age, gender, smoking, BMI, job seniority, lifting/carrying time at work, physical activity at work, and leisure, sitting with arms above 90°	Negative association of short workplace sitting bout with neck/shoulder pain intensity and positive association with moderated workplace sitting bout with neck/shoulder pain intensity. No association of prolonged Workplace sitting bout nor leisure-time sitting bouts with neck/shoulder pain intensity **Workplace sitting bout:*** Brief* Coefficient (B) = -0.38, OR(95%) = 0.60(0.40 – 0.91), *p* = 0.04; *Moderate* B = 0.28, OR(95%CI) = 1.23(0.93 – 1.63), *p* = 0.02; *Prolonged* B = − 0.08, OR(95%CI) = 0.84(0.69 – 1.02), p = 0.33. **Leisure sitting bout:*** Brief* B = 0.23, OR(95%CI) = 1.25(0.71 – 2.21), *p* = 0.44; *Moderate* B = 0.27, OR(95%CI) = 0.76(0.52 – 1.10), *p* = 0.15; *Prolonged* B** = **0.11, OR(95%CI) = 0.90(0.71 – 1.14), *p* = 0.37	0.91
**Study design – prospective**							
***Occupational Sedentary Behaviour***							
Hallman et al. 2016 [112] Denmark	All adults Duration: 12-months Sample size = 625 Average: age = 44.8(9.8), BMI = 27.5(4.9) %Female: 45% DPHACTO	Tradespeople – Cleaning, manufacturing, transport	Occupational – Total workplace sitting [2.4(1.7)hrs] Device-measured – ActiGraph	Neck/shoulder pain-intensity – 1-month prevalence (measured over 12 months) %Prevalence/incidence: 70%; mean pain score 3.1(2.7) Self-reported – Numerical rating scale (NRS)	Linear mixed models Adjusted for age, gender, and BMI; occupational sector, lifting/carrying time at work, physical activity at and leisure, working with the dominant arm elevated > 60°	Negative association of increased workplace sitting with neck/shoulder pain-intensity (i.e., reduced neck/shoulder pain-intensity) after 12-month follow-up in the Tradespeople Coefficient, B = 0.012, SE = 0.055, 95%CI = 0.000 – 0.025, *p* = 0.006	0.91
Korshøj et al. 2018 [39] Denmark	All adults Duration: 12-months Sample size = 665 Average: age = 45.0(10.0), BMI = 27.4(4.9) %Female: 44.2% DPHACTO	Tradespeople – Cleaning, manufacturing, transport	Occupational – Total workplace sitting, sitting bout Device-measured – ActiGraph	LBP-intensity – 3- and 12-month prevalence Mean pain score 3.1(2.7) Self-reported – Numerical rating scale (NRS), which ranges from 0 (‘no pain’) to10 (‘worst pain imaginable’)	Linear mixed models Adjusted for herniated disc, occupational lifting and carrying, LBP the last 3 months from baseline, sitting time during leisure time	Negative association of both total workplace sitting and temporal patterns of sitting (sitting bout) with LBP intensity across 12-month **Total workplace sitting:** Coefficient(B) = -0.050, SE = 0.007, p < 0.001, 95%CI = -0.065 – -0.040; Brief **(bouts ≤ 5 min):** B = -0.118, SE = 0.017, *p* < 0.001, 95%CI = -0.152 – -0.084; **Moderate (bouts of > 5 − 20 min):** B = -0.117, SE = 0.017, *p* < 0.001, 95%CI = -0.151 – -0.084; **Prolonged (bouts of > 20 min):** B = -0.123, SE = 0.018, *p* < 0.001, 95%CI = -0.158 – -0.088	0.95
Yip, 2004 [113] Hong Kong	All adults Duration: 12 months Sample size = 144 Average0: age = 31.1, BMI = NR %Female: 85.5%	Professionals – Nurses	Occupational – Workplace sitting (≥ 2 h) Self-reported	LBP – 12-month incidence %Prevalence: 56% Self-reported	Chi-square test	No association of prolonged workplace sitting ≥ 2 h/day with the prevalence of LBP, *p* = 0.47	0.59
***Non-occupational Sedentary Behaviour***							
Santos et al. 2020 [114] Brazil	All adults Duration: 24-months Sample size = 978 at baseline Average: age – median age(IQR) Baseline = 42(34 – 49), Follow-up = 44(36 – 51); BM – median BMI(IQR) Baseline = 25.2(22.8 – 28.2), Follow-up = 25.6(23.2 – 28.6) %Female: 66.6% baseline Pro-Mestre study	Professionals – Teachers	Non-occupational – TV time Self-reported	Chronic MSP – 6-month prevalence % Prevalence – baseline = 32.3%; follow-up = 24.7% Self-reported	Generalized estimating equation (GEE) regression Adjusted for age, sex, BMI, and depression	Positive association of change in TV time (30 min/day) with chronic MSP, OR(95%CI) = 1.051(1.001 – 1.102)	0.95
Jun et al. 2020 [115] Australia, South Korea	All adults Duration: 12-month Sample size = 214 (Australia – Brisbane = 156; South Korea – Daegu = 58) Average: age = 37.3(9.9), BMI = 24.0(4.2) %Female: 55.1%	Office workers – University faculty members, research centre, management service, industrial institution	Non-occupational – Total SB [total hours sitting in weekdays = 51.9(11.8)] Self-reported – IPAQ	Neck pain – monthly prevalence for the 12-month %prevalence/incidence: 18.2% self-reported	Survival analysis Adjusted for age, gender, and BMI	Positive association of increased total SB during weekdays with increased risk of neck pain Adjusted HR(95%CI) = 1.04(1.03 – 1.06), *p* < 0.001	0.82
***Occupational and Non-occupational Sedentary Behaviour***							
Lunde et al. 2017 [57] Norway	All adults Duration: 6-month Sample size = 124 Average: age – Construction = 39.9(13.6), Health = 44.5(9.6); BMI – Construction = 25.7(3.3), Health = 25.1(3.8) %Female – Construction = 1.6%, Health = 77.8%	Tradespeople – Construction; Professionals – Healthcare workers	Occupational – Total workplace sitting (Construction = 156.8(114.2) Health = 171.6(93.8); and Non-occupational – Leisure-time sitting (Construction = 282.0(78.4); Health = 274.0(94.3)) Device-measured – ActiGraph	LBP-intensity; 1-month prevalence %Prevalence: Health – Baseline = 59%; 6-month = 55%; Construction – Baseline = 52%; 6-month = 49%; mean pain score Baseline – Construction = 0.5(0.5); Health = 0.6(0.5); 6-months – Construction = 0.7(0.9); Health = 1.0(1.0) Self-reported	Linear mixed models Adjusted for age, gender, smoking, body mass index, heavy lifting, forward bending at work, social climate, decision control, fair leadership, empowering leadership, sitting (minutes) during leisure time	*Total full day Sitting:* Association of the total full day sitting with LBP-intensity in both healthcare and construction workers at baseline and 6-months Healthcare: **Baseline** – B(95%CI) = -0.16(-0.40 – 0.08), *p* = 0.183; **6-month** – B(95%CI) = -0.17(-0.40 – 0.07), *p* = 0.168 Construction: **Baseline** B(95%CI) = -0.07(-0.31– 0.18), *p* = 0.596; **6-months** – B(95%CI) = -0.08(-0.31– 0.17), *p* = 0.541 *Workplace Sitting* Healthcare workers – a negative association of workplace sitting with LBP intensity at baseline and 6-months’ follow-up **Baseline:** B(95%CI) = B(95%CI) = -0.31(-0.63 – 0.01), *p* = 0.058; **6-Month:** B(95%CI) = -0.34(-0.66 – -0.02), *p* = 0.040 Construction workers – no associations of workplace sitting with LBP intensity **Baseline:** B(95%CI) = -0.00001(-0.35 – 0.35), *p* = 1.00; **6-Month:** B(95%CI) = -0.003(-0.36 – 0.35), *p* = 0.986	0.95

^a^Measured multiple MSP conditions but presented only the MSP condition that was reported in the study result NR: Not reported, NMQ: Nordic musculoskeletal questionnaire, TV: Television-viewing,

**Table 3:** Characteristics of the experimental/intervention of occupational cohort studies

**Study ID + Country**	**Study design + Time points + Sample size + Intervention**	**Study population + Average age/ BMI + %Female**	**Sedentary behaviour (SB) domain + measures**	**Musculoskeletal pain (MSP) conditions + Time points/% prevalence + Measures**	**Statistical analysis + Adjusted covariates**	**Conclusions on associations of SB with MSP conditions + Effect Size/*p*-value**	**Quality score**
**Randomised controlled trial – RCT**							
Benzo et al. 2018 [116] USA	RCT Sample size = 15 Time points: 13 data points (minute 0, 10, 29, 60, 70, 89, 120, 130, 149, 180, 190, 209 and 240) – 4-h experiment	All adults – Office workers Average: age – 36.7(5.5), BMI = 29.6(3.1) %Female: 13.3%	Occupational – Sitting changes (sitting condition)	Physical MSP discomfort Incidence: average comfort scores 13 Self-reported – General Comfort Scale (GCS)	Linear mixed-effects (LME) regression Adjusted for age, gender, BMI, blood pressure	Positive association of 4 h of uninterrupted sitting with increased self-reported physical MSP discomfort, which was reduced with 10-min, hourly bouts of standing and pedalling	0.79
Brown et al. 2020 [117] Australia	RCT Sample size: AA = 32 (Control = 11; Intervention = 21) Time points: Baseline and 1-month follow-up Sit-stand workstations	All adults – Office workers Average: age = 43.0(1.8), BMI = 25.1 (4.0) %Female = 75%	Occupational – Usual sitting condition	MSP – Upper extremity (shoulders, elbows, hands); trunk (neck, upper back, lower back); lower extremity (hips, knees, ankles) and total body 7-days prevalence Self-reported – NMQ	Fisher’s exact test to evaluate between-group differences in MSP	Sitting reduction does not increase the risk of MSP compared to usual sitting at work	0.71
Coenen et al. 2017 [118] Australia	RCT Sample size = 201 (Intervention = 118; Control = 83) Time points: Baseline, 3-month Stand Up Victoria	All adults – Office workers Average: age – All = 45.3(9.3), Intervention = 44.8(8.9), Control = 46.1(9.7); BMI: NR %Female: All = 69%, Intervention = 65%, Control = 73%	Occupational – Sitting changes (sitting bout) Device-measured – activPAL	LBP, lower extremity symptoms, and upper extremity symptoms 7-day prevalence %Prevalence: At baseline LBP 52%, lower extremity 54%, and upper extremity 69% Self-reported – NMQ	Multivariable linear regression Adjusted for smoking, height, waist circumference, work productivity, mental demands at work, and fatigue	The intervention was effective in reducing workplace sitting time and increasing standing time The intervention was significantly effective by just over half an hour/day [34.6(0.9 – 68.3), *p* = 0.040] in individuals without LBP [MD95%CI = -126.6(-151.4 − 101.7), *p* < 0.001] than those with LBP [MD95%CI = -91.9-120.7 − 63.1), *p* < 0.001] Differences in intervention effect on extremities pain symptom were smaller and not statistically significant *Lower extremity:* [3.1(-28.8 – 35.0), *p* = 0.838]; *upper extremity:* [16.2(-28.3 – 60.7), *p* = 0.446] Prolonged sitting bout negatively association with extremities pain	0.88
Coenen et al. 2018 [33] Australia	Cross-sectional analysis of baseline dataset of RCT Sample size = 216 Stand Up Victoria	All adults – Office workers Average: age -45.4(9.3, BMI: NR %Female: 69%	Occupational – total workplace sitting time, sitting bout Device-measured – activPAL	LBP, lower-extremities, and upper-extremities 3-month prevalence %Prevalence: LBP = 68%, lower extremities = 69%, and upper extremities = 83% Self-reported – NMQ	Multivariable probity regression Adjusted for smoking, height, waist circumference, sitting not at work, standing not at work, stepping not at work, mental demands at work, and fatigue	No association of sitting time with LBP and extremities pain **Upper tirtle sitting time:*** LBP* – B = 0.01 (95%CI = -0.18 0.20), *p* > 0.999; *Lower extremities* – B = -0.05(-0.32 – 0.22), *p* = 0.934; *Upper extremitie*s – B = -0.08(-0.22 – 0.05), *p* = 3.28 No association of sitting bout with LBP but a negative association with extremities pain **Upper tirtle sitting bout:*** LBP* – B = -0.10(-0.29 – 0.09), *p* = 0.433; *Lower extremities* – B = -0.17(-0.34 – 0.01), *p* = 0.061; *Upper extremities* – B = -0.18(-0.34 – -0.02), *p* = 0.029	0.91
Danquah et al. 2017 [35] Denmark and Greenland	RCT Sample size = 317 (Intervention = 173; Control = 144) Time points: Baseline, 1-month, 3-month Take a Stand!	All adults – Office workers Average: age -All = 46(10), Intervention = 47(10) Control = 46(11); BMI: All = 26(4.9), Intervention = 26(5.0), Control = 27(4.8) %Female: All = 66%, Intervention = 61%, Control = 73%	Occupational –Sitting changes (sitting bout) Device-measured – ActiGraph	Neck/shoulder pain, low back pain, extremities as well as total pain score combining the degree of pain and number of pain sites 14-days incidence %Incidence: Neck/shoulder pain 51%, LBP 41% and extremities pain 38%; Average total pain score = 1.6(1.6) Self-reported	Multilevel mixed-effects logistic regression Adjusted for workplace, gender, and age	The intervention reduced workplace sitting time Sitting reduction positively associated with reduction in **neck/shoulder pain** [OR(95% CI) = 0.52(0.30 – 0.92), *P* = 0.02], but no significant association with reduction in in **LBP** [OR(95% CI) = 0.91(0.51 – 1.63), *p* = 0.74] and e**xtremities pain** [OR(95% CI) = 1.00(0.59 – 1.69), *p* = 0.99] Also, sitting reduction was significantly associated with **general MSP** score [B(95% CI) = -0.17(-0.32 – -0.01), *p* = 0.04]	0.83
E F Graves et al. 2015 [119] UK	RCT Sample size = 47 (Intervention = 26; Control = 21) Time points: At baseline, 4 weeks (mid-intervention) and 8 weeks (end-intervention) Sit-stand workstations	All adults – Office workers Average: age – All = 38.6(9.5), Intervention = 38.8(9.8), Control = 38.4(9.3); BMI – All = 24.8(4.4), Intervention = 24.9(4.4), Control = 24.7 ± 4.6 %Female: All = 79%, Intervention = 89%, Control = 67%	Occupational – Sitting changes Self-reported – Ecological Momentary Assessment (EMA)diaries	LBP, UBP, and neck/shoulder pain/discomfort Incidence at 4-weeks and 8-weeks during the intervention Self-reported – Likert scale from 0 (no discomfort) to 10 (extremely uncomfortable)	ANCOVA, Anthropometric, sociodemographic, work-related, and office-environment characteristics were potential confounders	Intervention beneficially reduced workplace sitting time The intervention did not increase musculoskeletal discomfort or pain Beneficial reductions in UBP and neck/shoulder pain/discomfort Adjusted Mean Difference(95%CI) **UBP** = -0.9 ( -1.9 – 0.2); **Neck/shoulder pain/discomfort** = -0.6 (-1.5 – 0.2) No significant benefit with reduction in LBP discomfort Adjusted Mean Difference(95%CI) = -0.2 (-1.0 – 0.7)	0.79
Renaud et al. 2020 [120] Netherlands	RCT Sample size = 244 Time points: Baseline, 4- month and 8-month follow-up Dynamic Work intervention – adjustable sit-stand workstations	All adults – Office workers Average: age – Intervention = 43.0(10.3), Control = 41.5(10.1); BMI: NR %Female: Intervention = 57.0%, Control = 62.6%	Occupational – Sitting changes Device-measured – activPAL	Neck/shoulder pain (Neck, shoulders, or upper back); Upper limbs pain (arms, wrists or hands); LBP; Lower limb pain (hips, thighs, knees, ankles, or feet) intensity 3-month prevalence Self-reported – NMQ (visual analogue scale (VAS) score)	Linear mixed and logistic mixed regression Adjusted for age, gender, and BMI	The intervention significantly reduced workplace sitting time at 4-month and 8-month **Total sitting, h/16 h:*** Baseline* – (*Control)* = 10.0 (1.2), *(Intervention)* = 10.1 (1.3); *4-month* – *(Control)* = 10.2 (1.2), *(Intervention)* = 10.2(1.3), OR(95% CI) = 0.11(0.43 – 0.22); *8-month* – *(Control)* = 10.2 (1.2), *(Intervention)* = 10.2(1.4), OR(95% CI) = 0.27(0.60 – 0.06) No significant association of workplace sitting time reduction with a reduction in musculoskeletal pain symptoms (intensity) at both 4-month and 8-month follow-up **Neck/shoulder pain:*** 4-month* – OR(95% CI) = 1.73(0.39 – 7.69); *8-month* – OR(95% CI) = 0.61(0.19 – 3.11). **Upper limbs:*** 4-month* – OR (95% CI) = 2.13(0.50 – 8.97); *8-month* – OR(95% CI) = 1.17(0.24 – 5.65). **LBP:*** 4-month* – OR(95% CI) = 0.97(0.40 – 2.38); *8-month* – OR(95% CI) = 0.53(0.19 – 1.43). **Lower limbs pain:*** 4-month* – OR(95% CI) = 0.44(0.07 – 3.00); *8-month* – OR(95% CI) = 0.20(0.02 – 1.87)	0.92
**Non-randomised controlled trial – Non-RCT**							
Brakenridge et al. 2018 [121] Australia	Randomised trial without control Sample size = 153 Time points: baseline, 3-, and 12-month Stand Up Lendlease	All adults – Office workers Average: age = 38.9(8.0), BMI = 24.6(3.4) %Female: 45.8%	Occupational – Sitting changes (mean sitting time 7.4(1.0)hr/10 h workday, prolonged sitting bouts ≥ 30 min reduction at work Device-measured – activPAL	Musculoskeletal symptoms – Neck, shoulder, elbow, wrists/hands, upper back, lower back, hips/thighs/buttocks, knees and ankle/feet 1-month prevalence %Prevalence: 79.3%; Mean pain scores: Lower extremity 0.7(1.1), upper extremity 0.7(1.0), LBP 1.4(2.0), neck 1.5(2.1), and total pain 1.1(1.1) Self-reported – NMQ	Mixed model Adjusted for age, sex, BMI category (normal/underweight, overweight/obese, missing), MVPA, mental quality of life, physical quality of life, job control score, work satisfaction score, desired sitting (over half/under half), current smoker (yes/no)	An hour of workplace sitting reduction is positively associated with significant small-to-moderate reductions in LBP [Coefficient, B(95% CI) = 0.84(1.44 – 0.25), *p* = 0.005 – study completers, and B(95% CI) = 0.61(1.22 – 0.01), *p* = 0.047 – multiple imputation analyses] An hour reduction in prolonged sitting is associated with reduction in LBP [B(95% CI) = -0.39(-0.79 – 0.00), *p* = 0.050] The associations of sitting reduction were not significant with a reduction in other musculoskeletal pain symptoms **Neck pain:*** Sitting reduction* – B(95% CI) = 0.14(-0.43 – 0.72), *p* = 0.626, *an hour reduction in prolonged sitting* – B(95% CI) = 0.07(-0.31 – 0.45), *p* = 0.715; **Lower extremity:*** Sitting reduction* – B(95% CI) = 0.07(-0.21 – 0.35), *p* = 0.611, *an hour reduction in prolonged sitting* – B(95% CI) = 0.01(-0.17 – 0.20), *p* = 0.873	0.96
Engelen et al. 2016 [122] Australia	Non-RT pilot study Sample size = 34 Time points: Baseline; 2-month Active design office buildings designed for health promotion and connectivity	All adults – Office workers Average: age = NR, BMI = NR %Female: 73.5%	Occupational – Sitting changes Self-reported	LBP-intensity/discomfort^a^ 2-month prevalence/ incidence Self-reported	Paired t-tests compared baseline and follow-up	The intervention resulted in 1.2 h/day less workplace sitting time (83 – 67%, *p* < 0.01), with sitting displaced largely by standing (9 – 21%, *p* < 0.01) A positive association of sitting reduction and reduced LBP, participants reported less LBP [t-test = -2.53, *p* < 0.01]	0.42
Foley et al. 2016 [123] Australia	Non-RT cross-over design Sample size = 88 Time points: Baseline, 4 weeks(end-intervention), and 7 weeks(follow-up) ABW environment	All adults– Office workers Average: age = 38.1, BMI = 25.7 %Female: 43%	Occupational – Sitting changes Device-measured – ActiGraph, activPAL Self-reported – Occupational Sitting and Physical Activity Questionnaire (OSPAQ)	LBP^a^ 7-days discomfort at 4 week and after 7 week follow-up Self-reported – NMQ	Linear mixed model; adjusted for age and gender, as well as measurement time points and laboratory effects	The intervention significantly (*P* < 0.01) resulted in 13.8% reduced sitting time and 10.7% increased standing time among workers Intervention was not associated with an increase in musculoskeletal discomfort despite the increased standing time Participants were twice as likely to report LBP at baseline compared with during the intervention [OR(95% CI) = 1.98(1.06 – 3.67)]	0.77
Gao et al. 2016 [124] Finland	Non-RCT Sample size = 45 (Intervention = 24; Control = 21) Time points: Baseline; 6-month Sit-stand workstations	All adults – Office workers Average: age = All = 43.7(10.7), Intervention = 47.8(10.8), Control = 39.0(8.5); BMI = All = 24.1(3.9), Intervention = 24(3.9), Control = 23.3(3.8) %Female: All = 75.6%, Intervention = 70.8%, Control = 81.0%	Occupational – Sitting changes; and Non-occupational – leisure-time sitting Self-reported	LBP-intensity(discomfort)^a^ 6-month prevalence and incidence Self-reported	ANOVA for testing the intervention effects and Spearman’s correlation coefficient for assessing the strength of the correlation	The intervention significantly resulted in decreased workplace sitting time by 6.7% (*p* = .048) and increased standing time by 11.6% (*p* < .001) **Sitting change:** Intervention – Baseline = 75.5 ± 15.9; 6-month = 68.9 ± 16.2. Control – Baseline = 76.0 ± 19.9; 6-month = 81.0 ± 11.9, The sitting reduction was significantly correlated with the increased standing time (*r* = -0.719, *p* < .001) Reduction in sitting time was significantly positively correlated with increased low back comfort, thus reduced LBP (*r* = 0.344, *p* = 0.024)	0.63
Kar & Hedge 2020 [125] India	Randomised controlled cross-over Sample size = 80 Time points: Baseline and end of the experiment (65 min)	Young adults -Students Average: age = 26.04(8.61), BMI = 22.53(4.13) %Female: 50%	Occupational – Workplace sitting (7.22(2.49)hrs/day) Self-reported	Musculoskeletal discomfort Baseline and end of the experiment (65 min) Self-reported – NMQ (15-item visual analog discomfort scale – VAS)	MANOVA Adjusted for gender	Pairwise comparisons revealed that mean musculoskeletal discomfort for the “Sit-Stand-Walk work condition” was significantly lower compared to the “Sitting work condition”, a statistically significant mean difference (MD95%CI) = -11.28(22.41 – 0.15) SE = 0.84, *p* = 0.045	0.79
Park & Srinivasan, 2021 [126] USA	Non-randomised experiment without control Sample size = 12 Time points: Baseline and post-exposure Sit-stand workstations	Young to middle-aged – Office workers Average: age – Male = 23.5 (3.1); Female = 3.3 (3.6) %Female = 50%	Occupational sitting – 2 h continuous sitting (prolonged sitting condition)	LBP/discomfort Pain intensity – Baseline = 6.3 (3.8)%; post-exposure = 18.8 (14.0)% Self-reported – VAS	Repeated-measure analysis of variance (RANOVA)	Prolonged sitting significantly increased LBP/discomfort (*p* = 0.009)	0.58
Thorp et al. 2014 [127] Australia	Randomised controlled cross-over Sample size = 23 SIT-condition and STAND-SIT condition – Over 5 consecutive workdays Sit or Stand @ WorkStudy	All adults – Office workers Average: age = 48.2(8), BMI = 29.6(4.1) %Female: 26.1%	Occupational – Sitting changes Device-measured – activPAL	Musculoskeletal symptoms – Neck, shoulder, elbow, hand/wrist, upper back, lower back, hip/thigh, knee, and ankle/foot 12-month prevalence and past 5-workday of the experimental condition %Prevalence: 60.9% 12-momth prevalence self-reported – NMQ	Linear and logistic mixed models; McNemar’s test to determine significant changes in the prevalence of musculoskeletal symptoms between experimental conditions Adjusted for order effects	Reducing sitting with 30 min standing break is positively associated with a reduction of LBP discomfort **LBP:** Mean difference (95% CI) = -31.8 (-62.8 – -0.9), *p* = 0.03) No significant association was reported in other body regions **Mean difference and 95%CI:*** Upper back* = +4.5(-23.5 – 32.6); *Neck* = +3.8(-17.3 – 24.9); *Shoulder* = +9.1(-7.5 – 25.6); *Elbow* = 0(− 4.5 – 4.5); *Wrist/hand* = -4.5(-17.8 – 8.7); Knee = -4.5(-24.4 – 15.3); *Hip* = -9.71(-35.1 16.9); *Ankle/feet* = -13.6(-32.5 – 5.2);	0.83
Waongenngarm et al. 2020 [128] Thailand	Non-randomised experiment without control Sample size = 40 Time points: Baseline and every 10 min until completion of the 4-h sitting period	20 – 45 years adults – Office workers Average: age = 29 (3.9), BMI = 21.1(1.7) %Female: 72.5%	Occupational – Sitting continuously for 4 h (Experimental condition)	Musculoskeletal discomfort – Neck, shoulder, elbow, wrist, upper back, low back, buttocks, hip/-thigh, knee, and ankle Baseline and every 10 min until completion of the 4-h sitting period Self-reported – Borg CR-10 scale (0 – 10 scale; 0 denotes no discomfort and 10 denotes extreme discomfort)	ANOVA to determine the effect of sitting time on perceived discomfort scores	Positive association of 4 h of continuous sitting with increased perceived musculoskeletal discomfort in all body regions. The body regions with the highest perceived discomfort were the low back, buttocks, upper back, thigh, and neck	0.64

^a^Measured multiple MSP conditions but presented only the MSP condition that was reported in the study result, NR: Not reported, NMQ: Nordic musculoskeletal questionnaire

In the general population category, SB was most frequently measured (79%) in the non-occupational domain. In contrast, in the occupational category, SB was most frequently measured (85%) in the occupational domain. Most (i.e., 54 out of 79) of the studies measured self-reported SB. In total, 19 studies investigated device-measured SB, including ActiGraph (general population category, four studies; occupational category, eight studies), activPAL (five – all in the intervention studies of occupational category), and both ActiGraph and activPAL (one intervention study of occupational category). Four studies in the experimental/intervention category, however, were based on pre-determined or usual workplace sitting conditions.

Among the studies that examined full-day SB or sitting, more than twice as many were in the general population category (15 studies) as were in the occupational category (seven studies). More studies in the occupational category examined workplace sitting (21 studies) and leisure-time sitting (seven studies) than in the general population category (workplace sitting time, two studies and leisure time, zero studies). Time spent watching television and/or other SBs were investigated in seven studies (six in the general population and one in the occupational cohort categories). Also, computer time (five studies) and vehicle time (five studies) were examined only in the occupational category. In addition to SB or sitting time, five studies examined SB/sitting bout duration, four of these studies were in the occupational category. Finally, 11 experimental/intervention studies examined changes in self-reported or device-measured sitting time.

Regarding MSP condition outcomes, 38 studies investigated a single MSP condition, 30 studies investigated multiple MSP conditions and 11 studies investigated general MSP. In general, LBP (50 studies) and neck/shoulder (28 studies) were the most frequently investigated. Except for two studies in the general population category that examined either medical record data or clinical examination data, all the studies investigated self-reported MSP conditions. In total, 22 studies investigated MSP-related pain intensity (19 studies) or MSP-related disability, and only three of these studies were in the general population category.

Regarding the population, 10 of 24 general population studies were of adults ≥ 45 years, including three studies of older adults (≥ 65 years). Also, one study in this category which was conducted in 2013 was of a 1946 birth cohort. In the occupational category, the studies were of adults ≥ 18 years; among these, five studies specifically recruited young or middle-aged adults, and one study was of a cohort of 21-year olds.

### Inter-rater reliability and quality assessment

There was 83.9% agreement between the two reviewers for including or excluding studies. Decisions on seven studies were made after consultation with the three senior reviewers.

Quality assessment scores for the studies are presented in Tables 1, 2, and 3 for the general population, observational-occupational, and experimental/intervention studies, respectively. On average, the studies in each of the categories were of high quality with mean scores of 0.83, 0.80, and 0.76 for the general population, observational-occupational, and experimental/intervention studies, respectively. The lowest scores in these categories were 0.41 for Aweto et al. [58], a cross-sectional study in the general population category; 0.36 for Omokhodion et al. [94], a cross-sectional study in the observational-occupational category; and 0.42 for Engelen et al. [122], a non-RT without control design pilot study in the experimental/intervention category. The highest score among the general population category was 0.95 scored in six studies [16, 64, 66, 67, 75, 76]. In the occupational category, the highest score in observational studies was 0.95 scored by six studies [39, 57, 100, 109, 114, 129], and in experimental/intervention studies was 0.96 for one study, Brakenridge et al. [121].

The low-quality studies mostly scored low for QualSyst checklist item-11, “Some estimate of variance is reported for the main results?”. Most of the experimental/intervention studies scored low on item 9, “Sample size appropriate?”. In general, most of the studies scored average on item 8, “Outcome and (if applicable) exposure measure(s) well defined and robust to measurement/misclassification bias? Means of assessment reported?”. Overall, based on a relatively liberal cut-off threshold of 0.55 put forward by Kmet & Lee [52], six studies scored ≤ 0.55 (general population two, observational-occupational three, and experimental/intervention occupational one); when based on a relatively conservative 0.75 cut-off threshold, 56 studies scored > 0.75 (general population 18, observational occupational 28 and experimental/intervention occupational 10). Studies that scored above 0.75 were considered high-quality, and those that scored below were considered low-quality studies.

### Associations of non-occupational sedentary behaviour with musculoskeletal pain conditions

Table 4 shows the key associations of non-occupational SB with MSP conditions and Table 5 summarises the findings.

**Table 4:** Summary of key associations of sedentary behaviour with musculoskeletal pain conditions by studies quality

**Sedentary Behaviour**	**MSP Conditions**	**Cross-Sectional Studies**									**Prospective Studies**								
		**Positive Association**			**No Association**			**Negative Association**			**Positive Association**			**No Association**			**Negative Association**		
		***Quality***			***Quality***			***Quality***			***Quality***			***Quality***			***Quality***		
		**All**	**High**	***Low***	***All***	***High***	***Low***	***All***	***High***	***Low***	***All***	***High***	***Low***	***All***	***High***	***Low***	***All***	***High***	***Low***
***Non-occupational sedentary behaviour***^***b***^		***Observational studies***																	
Total SB	LBP	**5**	4	1	**4**	3	1	**1**	1		**1**		1	**1**	1				
	LBP-intensity	**1**	1											**1**	1				
	Neck/shoulder pain	**1**	1																
	Neck/shoulder pain-intensity				**1**	1													
	Knee pain	**4**	4											**1**	1				
	Arthritis	**2**	2		**1**	1													
	Hip pain	**1**	1					**1**	1										
	Extremities pain				**1**	1													
	General MSP	**2**	2		**2**	1	1				**1**		1						
Total SB bout	LBP^c^	**1**	1																
	LBP-intensity^d^	**1**	1					**1**	1										
SBs/TV time	LBP	**1**		1	**2**	1	1												
	LBP-intensity													**1**	1				
	LBP-disability^e^										**1**	1							
	Neck/shoulder pain				**2**	1	1												
	Back/neck pain				**1**	1													
	Knee pain	**1**		1															
	Extremities pain	**1**		1	**1**	1													
	General MSP	**1**	1								**1**	1							
Leisure-time SB	LBP	**1**		1	**1**	1													
	LBP-intensity	**2**	2		**1**	1													
	Neck/shoulder pain				**1**		1												
	Neck/shoulder pain-intensity	**1**	1																
	Extremities pain				**1**		1												
***Occupational sedentary behaviour***		***Observational studies***																	
*Workplace sitting*																			
Device-measured	LBP				**1**	1													
	LBP-intensity^f^				**2**	2								**1**	1		**2**	2	
	Neck/shoulder pain-intensity	**1**^** g**^	1		**2**	2											**1**	1	
Workplace sitting bout^a^	LBP-intensity	**1**	1					**1**	1								**1**	1	
Self-reported	LBP	**7**	5	2	**4**	4								**1**		1			
	Neck/shoulder pain	**9**	6	3	**1**	1					**1**	1							
	Knee pain																**1**	1	
	Extremities pain	**2**	1	1	**4**	3	1	**3**	2	1									
Computer time	LBP	**1**	1					**1**	1										
	Neck/shoulder pain	**2**	1	1															
	General MSP	**1**		1	**1**	1													
Vehicle time	LBP	**2**	1	1	**1**	1													
	General MSP	**1**		1	**1**	1													
**Occupational sedentary behaviour**		**Experimental/intervention studies**																	
*Changes in sitting time*		***Randomised controlled trial***									***Non-randomised controlled trial***								
Sitting reduction	LBP/discomfort	**1**	1		**2**	2					**5**	3	2						
	Neck/shoulder pain/discomfort	**2**	2		**1**	1								**2**	2				
	Extremities pain				**4**	4								**2**	2				
	General MSP/discomfort	**2**	1	1							**1**	1							
Prolonged sitting	General MSP/discomfort	**1**	1								**1**		1						
	LBP/discomfort										**1**		1						
Prolonged sitting bout	Extremities pain							**1**	1										

Numbers in the Table represent the number of studies

*LBP:* Low back pain, *MSP:* Musculoskeletal pain, TV: Television-viewing, *SB(s):* Sedentary behaviour(s) including sitting watching television, video game, reading, listening to music, talking on the phone

^a^A negative association for a moderate sitting bout and a positive association for a brief bout in the cross-sectional study

^b^Included both self-reported and device-measured occupational SB

^c^A positive in association obese individuals

^d^Positive association in normal-weight individuals and a negative association in overweight/obese individuals

^e^Association in females but not in males

^f^One study reported no association in construction workers and a negative association in healthcare workers

^g^Low SB negatively associated with neck/shoulder pain-intensity in men but not women, thus high SB probably increase the risk of neck/shoulder pain-intensity in men

**Table 5:**
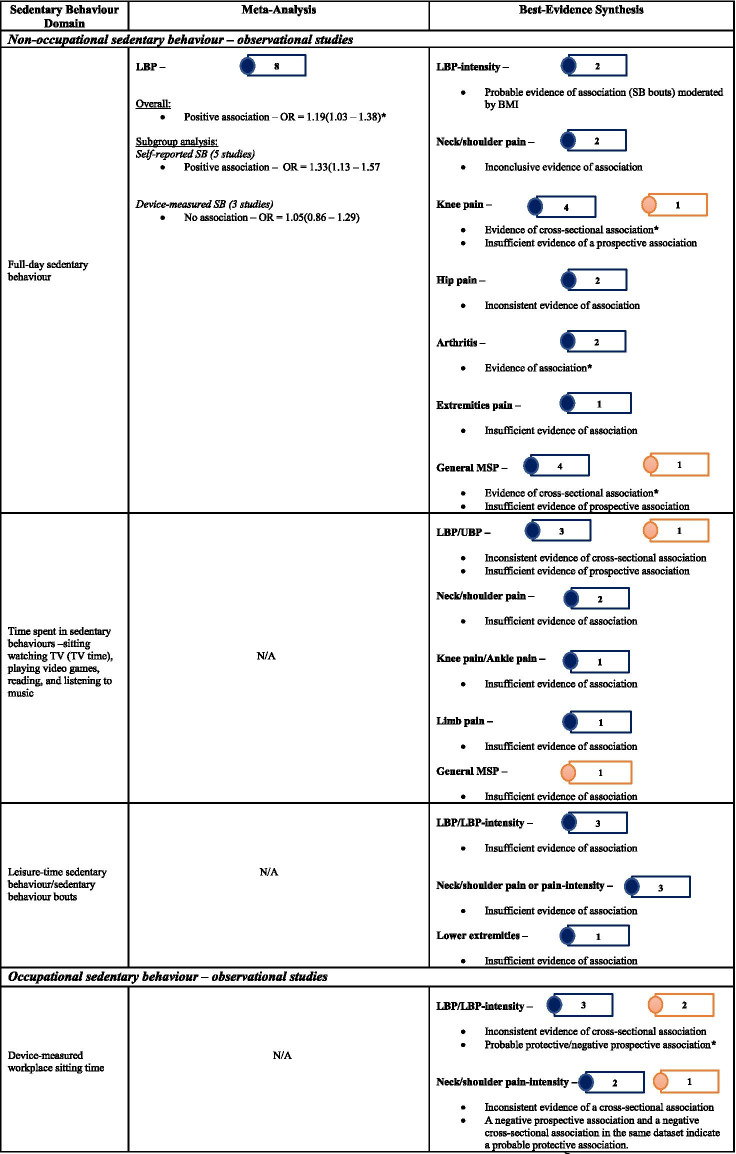

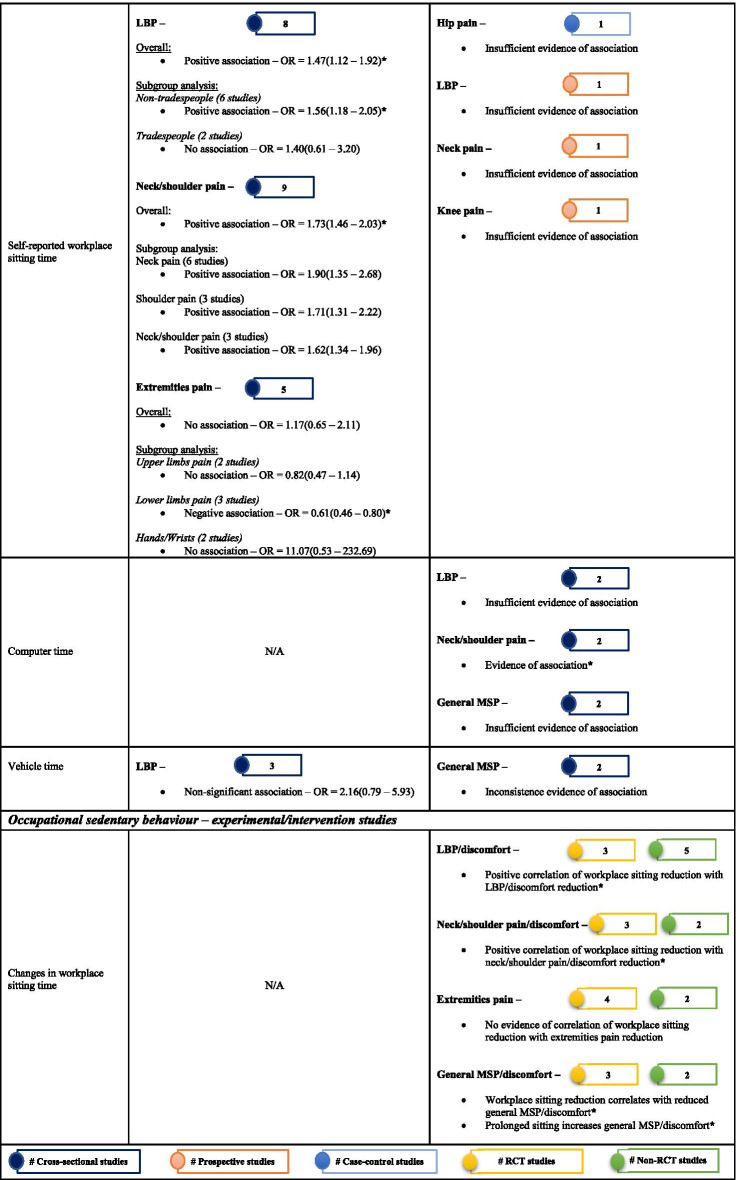
Summary of findings synthesised by meta-analysis and the best-evidence synthesis

The numbers in the box indicate the number of studies considered in the evidence synthesis. The effect sizes in the meta-analysis indicate odds ratio with confidence intervals in brackets

*LBP:* Low back pain, *UBP:* Upper back pain, *MSP:* Musculoskeletal pain, *OR:* Odds ratio, *SB:* Sedentary behaviour, *RCT:* Randomised control trial, *TV:* Television-viewing, *BMI:* Body mass index, *N/A:* Not Applicable due to variations in included studies

^a^The key findings

#### Full-day sedentary behaviour or sitting time

##### Low back pain

**Fig. 2:**
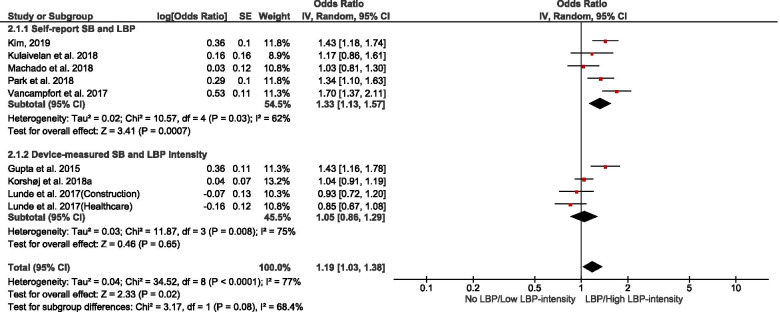
A forest plot for inverse-variance meta-analysis using a random effect of cross-sectional studies that investigated non-occupational sedentary behaviour (SB) showing the pooled effect size of the association of full-day SB with low back pain (LBP); subgroup analysis by self-reported SB and LBP and device-measured SB and LBP-intensity

Fourteen studies in total (10 general population [59–61, 63, 66, 68–70, 75, 77] and four occupational [57, 108, 109, 129]) examined the association of full-day SB/sitting time with LBP [59–61, 63, 66, 68–70, 75, 77] or LBP-intensity [57, 109, 129], including two studies [69, 129] that also examined full-day SB bout. Among these studies, 11 were cross-sectional [57, 59–61, 63, 66, 68–70, 108, 109, 129] and three applied a prospective [57, 75, 77] design; one study [57] reported both cross-sectional and prospective analyses. In the cross-sectional studies, six reported a positive association [60, 66, 68–70, 109] and four reported no association [59, 61, 63, 108, 129]. Five of the positive association studies [60, 66, 69, 70, 109] and three with no associations [57, 59, 129] were of high quality. Further, one of the two high-quality cross-sectional studies that investigated full-day SB/sitting bout reported a positive association in obese individuals [69]; whereas the other study [129] reported a positive association in non-overweight individuals (BMI < 25kgm^−2^), and a negative association in overweight/obese individuals (BMI ≥ 25kgm^−2^). This suggests probable evidence of cross-sectional association of full-day SB/sitting bout with LBP-intensity which is moderated by BMI. Eight of these cross-sectional studies were considered in a meta-analysis, including five studies [60, 61, 63, 66, 70] that investigated self-reported full-day SB and LBP and three studies [57, 109, 129] that analysed device-measured full-day SB/sitting and LBP-intensity (Fig. 2). The overall pooled effect size indicated full-day SB is positively associated with LBP [OR = 1.19(1.03 – 1.38), *p* = 0.02], though a significantly moderate-high heterogeneity was observed (I^2^ = 77%, *p* < 0.00001). A subgroup analysis by self-reported and device-measured full-day SB showed a cross-sectional association of self-reported full-day SB with LBP [OR = 1.33(1.13 – 1.57), *p* = 0.007; I^2^ = 62%, *p* = 0.03], but no association of device-measured full-day SB/sitting with LBP-intensity in mostly tradespeople [OR = 1.05(0.86 – 1.29), *p* = 0.65; I^2^ = 75%, *p* = 0.008]. The robustness of the analysis was tested in a sensitivity analysis (Supplementary Figure 1A) by excluding two studies [61, 63] with low-quality; the overall and the self-reported full-day SB subgroup associations remained significant.

For the prospective studies, the evidence was inconsistent with a positive association of full-day SB with LBP reported in one low-quality study [77], and two high-quality studies reported no association of self-reported full-day SB [75] and device-measured [57] full-day sitting with LBP [75] and LBP-intensity [57] respectively.

##### Neck/shoulder pain

There were two high-quality cross-sectional studies [64, 110] that investigated the association of device-measured full-day SB with neck/shoulder pain-intensity [110] and shoulder pain [64]. One study [110] of tradespeople reported a positive association of high full-day SB with neck/shoulder pain-intensity. The other study [64] of severely obese individuals reported no association of low full-day SB with shoulder pain, which may imply a high full-day SB could be associated with shoulder pain. Thus, there is inconclusive evidence of a cross-sectional association of full-day total SB with neck/shoulder pain.

##### Knee/hip pain/arthritis

Four high-quality cross-sectional studies, three of adults ≥ 45 years in the general population cohorts [16, 59, 60] and one study of adults < 40 years in the occupational cohorts [105] reported a positive association of full-day SB with knee pain (osteoarthritis), including one study that reported the association only in men [59]. There was one prospective study [76] that reported no association of extensive full-day SB with knee pain. According to the best-evidence synthesis, we concluded there is strong evidence of cross-sectional association of full-day SB with knee pain in middle-aged to older adults, however, there is insufficient evidence whether the association is gender-dependent. Also, there is insufficient evidence of a prospective association of full-day SB with knee pain. Also, of the two high-quality cross-sectional studies [60, 64], one reported a positive association of self-reported full-day SB with hip pain [60], and the other a positive association of device-measured low full-day SB with hip pain, indicating a protective association of high full-day SB with hip pain [64]. Therefore, there is insufficient evidence of a cross-sectional association of full-day SB with hip pain. Furthermore, two high-quality cross-sectional studies [62, 70] in the general population category reported a positive association of full-day SB with arthritis of adults ≥ 50 years [70] or ≥ 65 years [62] old. Another high-quality cross-sectional study [60], however, reported no association of full-day SB with osteoarthritis of adults ≥ 65 years old. Thus, there is evidence of a cross-sectional association of full-day SB with arthritis in adults ≥ 50 years.

##### Extremities pain

One high-quality cross-sectional study in the general population cohort reported an association of wrist/hand pain with a high volume of full-day SB, but no association with a low volume of full-day SB [64]. However, evidence in one study is insufficient to conclude.

##### General musculoskeletal pain

Four cross-sectional studies investigated full-day SB and general MSP. Two high-quality studies of the general population category reported a positive association [65, 67] and two studies (one high-quality [106] and one low-quality [107]) study of the occupational category reported no association. Based on the high-quality studies, there is strong evidence of a cross-sectional association of full-day SB with general MSP. However, the evidence of a prospective association is inconclusive with only one low-quality study in the general population category reporting a positive association [17].

#### Time spent in sedentary behaviours – sitting watching TV, video games, reading, listening to music

Five cross-sectional [58, 61, 72, 73, 86] and two prospective [78, 114] studies – five of general population [58, 61, 72, 73, 78], two of occupational [86, 114] – investigated time spent in SBs and MSP conditions [58, 61, 72, 73, 86] or MSP-related outcomes [78]. Three were of high-quality [72, 73, 86] and two low-quality [58, 61]. There were variations in the MSP condition outcomes, hence meta-analysis was not performed for these studies. Among the cross-sectional studies, only one study [58] (low-quality) reported positive associations of SBs > 3 h/day with LBP, UBP, knee pain, and ankle pain, and no associations with neck/shoulder pain and elbow pain. Another study [86] (high-quality) also reported a positive association of TV-viewing time (TV time) > 2 h/day with clinically diagnosed MSP condition of > 50 year old adults. Most of the cross-sectional studies reported no associations of TV time (≥ 2 or 3 h/day) with LBP [61, 72], neck/shoulder pain [73], back/neck pain [86], or limb pain [86]. Based on the best evidence, there is insufficient evidence of cross-sectional associations of SBs or TV time with MSP conditions.

For the two prospective studies, both of high quality, one reported no association of TV time > 2 h/day with LBP-intensity, but a positive association with LBP-disability only in women [78]. The other study [114], however, reported a positive association of TV time with general MSP. Herein also, prospective evidence of associations of TV time with MSP conditions and MSP-related outcomes are insufficient.

#### Leisure-time sedentary behaviour

Five cross-sectional studies (four high-quality [104, 109–111] and one low-quality [56]) of occupational category examined the associations of self-reported [56, 104] and device-measured leisure-time SB [109–111] or SB bout [111] with LBP [56, 104], LBP-intensity [109], neck/shoulder pain [56], neck/shoulder pain-intensity [110, 111] and lower extremities pain [56]. All these studies except one [104] were of tradespeople, and two were from a single large study – “Danish PHysical ACTivity cohort with Objective measurements (DPHACTO) [110, 111]. Three of the studies reported a positive association of leisure-time SB with LBP [56], LBP-intensity [56, 109], and neck/shoulder pain-intensity [110], whereas three studies reported no association of SB [56, 104] or SB bout [111] with LBP [104], neck/shoulder pain [56], neck/shoulder pain-intensity [111] or lower extremities pain [56]. Based on the best-evidence synthesis, there is insufficient evidence of cross-sectional associations of leisure-time SB or SB bout with LBP, LBP-intensity, neck/shoulder pain, neck/shoulder pain-intensity, or lower extremities pain.

### Associations of occupational sedentary behaviour with musculoskeletal pain conditions

Table 4 (above) shows the key associations of occupational SB with MSP conditions and Table 5 summarises the findings.

#### Device-measured workplace sitting time

##### Low back pain

Three high-quality cross-sectional [33, 109, 129] and two high-quality prospective [39, 57] studies investigated device-measured workplace sitting [39, 57, 109, 129] or sitting bout [129] and LBP [33] or LBP-intensity [39, 57, 109, 129], including a study with both a baseline cross-sectional and a prospective analysis [57]. Two of these studies [39, 129] were from a single large study. One study was of office workers [33] and four studies were of tradespeople [39, 57, 109, 129], which included one study also with healthcare workers [57]. No association was reported in any of the cross-sectional studies, except one that reported a marginally significant positive association with LBP-intensity [109]. One cross-sectional study [129], nonetheless, reported a negative association of total workplace sitting or a moderate sitting bout with LBP-intensity in overweight/obese individuals (BMI ≥ 25kgm^−2^), and a positive association of brief bout workplace sitting with LBP-intensity in non-overweight individuals (BMI < 25kgm^−2^). The baseline cross-sectional analysis of one prospective study [57] reported a negative association with LBP-intensity in healthcare workers but no association in construction workers (tradespeople). Meta-analysis was not feasible, hence, the best-evidence synthesis indicates there is insufficient evidence of cross-sectional associations of device-measured workplace sitting with LBP and LBP-intensity in tradespeople and non-tradespeople. For the prospective studies, there were two high-quality studies [39, 57]; the association was inconsistent in one study with a reported negative association with LBP-intensity in healthcare workers but no association in construction workers [57]. The other study of tradespeople, however, reported a negative association of both total workplace SB and SB bout with LBP-intensity [39]. There is, therefore, an indication that sitting at the workplace may have a protective effect which is dependent on occupation type.

##### Neck/shoulder pain

Two cross-sectional studies [110, 111] and one prospective [112] study all from a single large study (all high-quality) examined the association of device-measured total workplace sitting or sitting bout with neck/shoulder pain-intensity of tradespeople. No association of high total workplace sitting with neck/shoulder pain-intensity was reported in the cross-sectional studies [110, 111]. One cross-sectional study [110], however, reported a negative association of low total workplace sitting with neck/shoulder pain-intensity in males but no association in females. Also, the other cross-sectional study [111] reported equivocal associations of workplace sitting bouts with neck/shoulder pain-intensity; a positive association for a moderate bout, and a negative association for a brief bout. A negative association was reported in the prospective study [112]. The cross-sectional association is inconsistent [110, 111], however, a negative association in a prospective analysis [112] of the same DPHACTO study dataset suggests there is a probable protective association of workplace sitting exposure with neck/shoulder pain-intensity in tradespeople.

#### Self-reported workplace sitting time

There were 19 cross-sectional [71, 82–89, 92–94, 96–98, 100–103], one case–control [74] and three prospective [79, 113, 115] studies that investigated self-reported workplace sitting and MSP conditions – LBP [71, 83, 87–89, 92, 94, 96–98, 102, 103, 113], neck/shoulder pain [82–85, 87, 88, 92, 93, 100, 103, 115], knee/hip pain [74, 79, 93] and extremities pain [83–86, 88, 92, 101]. All but three of these studies [71, 74, 79] were in the occupational category. The durations of the workplace sitting examined varied across the studies, included 20 min continuous [93], > 4.2 h/week [92], ≥ 2 h/day [74, 79, 87, 88, 113], ≥ 3 h/day [94], ≥ 4 h/day [82–85, 92, 97, 100, 103], ≥ 6 h/day [98], 51.9(11.8)hrs per total weekdays [115], or unspecified durations (prolonged sitting) [71, 86, 89, 96, 101, 102].

**Fig. 3:**
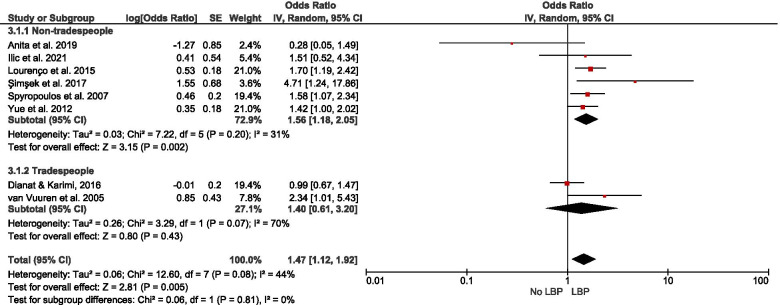
A forest plot for inverse-variance meta-analysis using a random effect of cross-sectional studies that investigated occupational SB showing the pooled effect sizes for the association of self-reported workplace sitting time with LBP; subgroup analysis by non-tradespeople and tradespeople

For the cross-sectional studies, of the 11 studies (two of office workers, five of professionals, and three of tradespeople, as well as one general population study) that examined associations with LBP, seven reported positive associations [92, 94, 96–98, 102, 103] and four reported no association [71, 87–89]. All these studies except two [94, 96] were of high-quality. Eight studies (all high-quality) were meta-analysed with a subgroup analysis according to non-tradespeople (office workers [98], professionals [89, 92, 97, 103], and general population [71]) and tradespeople [87, 102] as indicated in Fig. 3. Overall, there is a significant cross-sectional association of workplace sitting with LBP (OR = 1.47(1.12 – 1.92), *p* = 0.005; however, there is non-significant moderate heterogeneity (I^2^ = 44%, *p* = 0.08). The subgroup analysis indicates the association is significant in the non-tradespeople [OR = 1.56(1.18 – 2.05), *p* = 0.002] with moderate but non-significant heterogeneity (I^2^ = 31%, *p* = 0.20), and non-significant association in the tradespeople [OR = 1.40(0.61 – 3.20), *p* = 0.43] with substantial non-significant heterogeneity (I^2^ = 70%, *p* = 0.07). Sensitivity analysis (Supplementary Figure 2A) excluded two studies [71, 102] with lower quality score and the overall association as well as the association for non-tradespeople were still significant, and zero heterogeneity in the non-tradespeople (I^2^ = 0%).

**Fig. 4:**
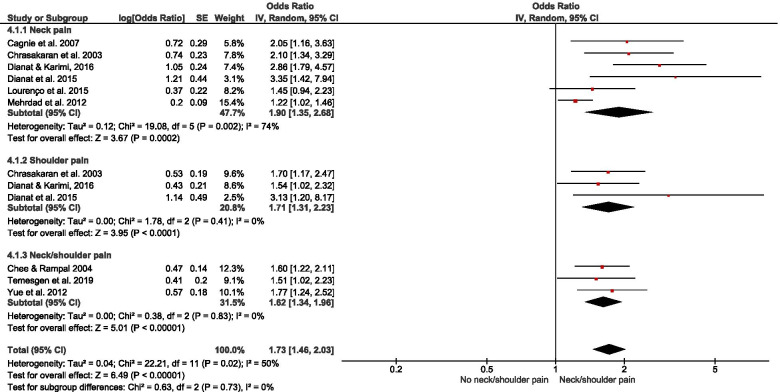
A forest plot for inverse-variance meta-analysis using a random effect of cross-sectional studies that investigated occupational SB showing the pooled effect size for the association of self-reported workplace sitting time with neck/shoulder pain; subgroup analysis by studies that reported neck pain, shoulder pain, and neck/shoulder pain

With neck/shoulder pain, a positive association was reported in eight studies (one of office workers [82], three of professionals [87, 88, 93, 100, 103], and four of tradespeople [84, 85]). Only one study [92] of professionals reported no association. Also, one study [83] reported a negative association only in females. Seven of these studies [84, 87, 88, 92, 93, 100, 103] were of high-quality. A meta-analysis (Fig. 4) of pooled effect sizes of nine studies [82, 84, 85, 87, 88, 92, 93, 100, 103] indicates workplace sitting is associated with increased odds of neck/shoulder pain [Overall OR = 1.73(1.46 – 2.03), *p* < 0.00001]. Subgroup analysis also shows there is increased odds of neck pain [OR = 1.90(1.35 – 2.68), *p* = 0.0002], shoulder pain [OR = 1.71(1.31 – 2.22), *p* < 0.0001] and neck/shoulder pain [OR = 1.62(1.34 – 1.96), *p* < 0.00001]. The overall heterogeneity was, however, significantly substantial (I^2^ = 51%, *p* = 0.02), mainly due to heterogeneity in studies on neck pain (I^2^ = 74%), as studies on shoulder and neck/shoulder pain were homogeneous (I^2^ = 0%). Sensitivity analysis (Supplementary Figure 3A) after excluding two studies [82, 85] with low-quality shows the estimate is robust and the association remained significant.

**Fig. 5:**
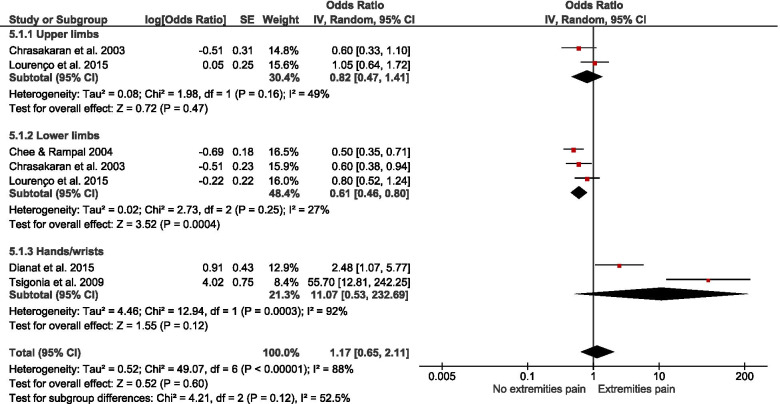
A forest plot for inverse-variance meta-analysis using a random effect of cross-sectional studies that investigated occupational SB showing the pooled effect size for the association of self-reported workplace sitting time with extremities pain; subgroup analysis by upper limbs, lower limbs, and hand/wrist pains

For extremities pain, a positive association with hand/wrist pain was reported in two studies [88, 101]; three studies [83, 84, 86] reported a negative association, including one study [83] with the association only in females; and another study [86] with lower limb disability; no association was reported in four studies [85, 87, 88, 92]. Five of the studies were of high quality. A pooled analysis (Fig. 5) of five studies [84, 85, 88, 92, 101] with considerable heterogeneity (I^2^ = 88%, *p* = 0.00001) indicated no association of workplace sitting with extremities pain [OR = 1.17(0.65 – 2.11), *p* = 0.60]; however, a subgroup analysis of three studies [84, 85, 92] with low and non-significant heterogeneity (I^2^ = 28%) indicated an inverse association of workplace sitting with lower limbs pain [OR = 0.61(0.46 – 0.80), *p* = 0.0004]. Sensitivity analysis shows the overall effect size remained non-significant (Supplementary Figure 4A).

The only case–control study [74] of the general population reported a positive association of workplace sitting with hip pain, insufficient evidence of association from a single study.

For the prospective studies, one of low-quality reported no association of workplace sitting with LBP [113]; another one of high-quality reported a positive with neck pain [115]; the third study of high-quality reported a negative association with knee pain [79]. Therefore, prospective evidence of association of workplace sitting is insufficient with LBP, neck pain, and knee pain.

#### Computer time

Five cross-sectional studies of the occupational category (office workers [80, 82] and professionals [86, 97, 103]), including three high-quality investigated computer time and LBP [97, 103], neck/shoulder pain [82, 103] or general MSP [80, 86]. A positive association of computer time ≥ 4 h/day was reported with LBP [97], neck/shoulder pain [82, 103], and general MSP [80], and a negative association reported with LBP in another study [103]. Also, one study reported no association of computer time ≥ 2 h/day with general MSP [86]. There is moderate evidence of a cross-sectional association of computer time with neck/shoulder pain, however, the evidence is restricted to a small number of studies. The evidence with LBP and general MSP is insufficient with limited studies.

#### Vehicle time

**Fig. 6:**
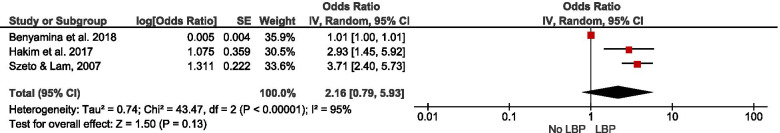
A forest plot for inverse-variance meta-analysis using a random effect of cross-sectional studies that investigated occupational SB showing the pooled effect size for the association of vehicle time with LBP

Five occupational category cross-sectional studies of bus drivers [90, 95, 99] and professionals (patrol duty police officers) [81, 91] reported vehicle time and LBP [81, 90, 99] or general MSP [91, 95], including three of high-quality [81, 91, 99]. There is an inconsistent association with general MSP; of the two studies [91, 95], one reported no association [91] and the other a positive association [95]. A similar inconsistent association was reported with LBP; two studies [90, 99] reported a positive association and one study [81] reported no association. In a meta-analysis (Fig. 6), the pooled effect size of the three studies [81, 90, 99] showed considerable heterogeneity (I^2^ = 95%) but increased odds of LBP with prolonged sitting in a vehicle, although this was not statistically significant [OR = 2.16(0.79 – 5.93), *p* = 0.13]. After excluding the low-quality study [90] in a sensitivity analysis the association was still non-significant (Supplementary Figure 5A).

#### Changes in workplace sitting time

Fourteen experimental/intervention studies investigated changes in sitting time and MSP symptoms, including LBP, neck/shoulder pain, extremities pain, and general MSP/discomfort of office workers [35, 116–124, 126–128] and students [125]. Designs included six RCTs [35, 116–120], two randomised controlled cross-over trial [125, 127], two non-randomised experiment without control [126, 128], one study each of non-RCT [124], RT without control [121], non-randomised cross-over trial [123], and non-RT pilot study.

Duration of experiments/interventions ranged from 65 min [125] to 12 months [121]. Sample sizes ranged from 12 participants [126] to 317 participants [35]. Nine of the studies were of high quality [35, 116, 118–121, 123, 125, 127] and four of low quality [117, 122, 124, 128]. Of the studies, nine measured sitting time change and reported a reduction in sitting time after the period (device-measured – ActiGraph [35, 123] and activPAL [118, 120, 121, 123, 127]; self-report [119, 122–124]) while three studies were based on fixed sitting duration (65 min [125] and 4 h [116, 128]), over 2-h continuous sitting [126] or usual work sitting condition [117].

There were methodological and analytical variations among the studies, therefore, the data were not meta-synthesised. A positive correlation of sitting reduction with a reduction in LBP was reported in six studies [118, 121–124, 127] (including four high-quality studies with one RCT [118]); reduction in neck/shoulder pain two RCT studies [35, 119] (both high-quality). No study reported a correlation or association of sitting reduction with a reduction in extremities pain. Two high-quality RCT studies [35, 120], however, reported no significant correlation with LBP; three studies [120, 121, 127] with neck/shoulder pain, all high-quality with one RCT [120]. Furthermore, of six studies, two high-quality studies [35, 125] reported sitting reduction correlates with a reduction in general MSP/discomfort; one RCT study [117] of low-quality reported reduced workplace sitting time does not increase the risk of general MSP/discomfort; and three studies [116, 126, 128], one of high-quality [116], reported a positive association of continuous uninterrupted sitting with increased general MSP/discomfort [116, 128] and LBP/discomfort [126]. Also, one high-quality study [118], however, reported a protective association of prolonged workplace sitting bout with extremities pain.

Generally, the best evidence suggests workplace sitting reduction is correlated with reduced LBP and general MSP symptoms. For neck/shoulder pain reduction, the evidence from RCT suggests there is a positive correlation with reduced workplace sitting. Also, there is moderate evidence of association of prolonged uninterrupted sitting with general MSP/discomfort. There is, nevertheless, no evidence of correlation of reduced workplace sitting with a reduction in extremities pain.

### Risk of bias

Three studies had lower quality scores detected by the QualSyst checklist, one of which was a pilot study and had a potential risk of bias; however, most of the studies did not show any major risk of bias. The funnel plots (Supplementary Figures 1B, 2, 3, 4 and 5B) of the meta-synthesised studies were mostly asymmetrical; this could be because of the small number of studies available and not likely due to publication bias. Also, the significant heterogeneity observed may have risen from the studies’ methodological heterogeneity in the variables measured and study sample.

## Discussion

### Key findings

This is the first review to examine separately the associations of occupational and non-occupational SB with MSP conditions in adults. We found in the non-occupational SB domain, strong evidence of cross-sectional associations for full-day SB with MSP conditions, including LBP, knee pain, arthritis, and general MSP. For the occupational SB domain, there is strong evidence of cross-sectional associations of self-reported workplace sitting with MSP conditions, including LBP and neck/shoulder pain. Also, we found moderate evidence of a cross-sectional association of computer time with neck/shoulder pain. Furthermore, we identified from experimental/intervention studies that reduced occupational sitting time was associated with a reduction in LBP, neck/shoulder pain, and general MSP. However, there was insufficient evidence on cross-sectional associations of leisure-time SB and TV time with MSP conditions. Likewise, the evidence on prospective associations of occupational and non-occupational SB with MSP conditions was insufficient, nonetheless, there is an indication that device-measured total workplace sitting could be negatively associated with LBP-intensity in tradespeople.

### Non-occupational sedentary behaviour and musculoskeletal pain conditions

We observed in our meta-analysis of cross-sectional studies that full-day SB or sitting time is positively associated with the risk of LBP. However, subgroup analysis by self-reported and device-measured SB indicated the association exists between self-reported full-day SB and LBP, but not for device-measured full-day SB and LBP-intensity, which included studies of mostly tradespeople. This finding is, nonetheless, limited by a small number of studies. The cross-sectional design and self-reported data downgrade the quality in this evidence with the association only present in the case of self-reported SB, but not device-measured SB, with LBP. Our narrative synthesis based on the best-evidence synthesis found that there are cross-sectional associations for full-day SB with knee pain, arthritis, and general MSP, but an inconclusive association with neck/shoulder pain. We found inconsistent cross-sectional associations of full-day SB with hip and extremities pains. Also, limited by the number of studies, there was insufficient evidence of prospective associations of full-day SB with MSP conditions. Furthermore, we observed inconsistent evidence of cross-sectional and prospective associations of SBs, TV time, and leisure-time SB with MSP conditions. These findings were, however, constrained by the limited number of studies available, especially evidence from prospective studies.

Our cross-sectional findings for LBP are in contrast to a previous review of observational prospective and case–control studies by Chen and colleagues, that showed no associations of a sedentary lifestyle with the risk of LBP [19]. Unlike our review which included only adults, Chen and colleagues’ review included both children and adults [19]. Another review of prospective studies has also reported some inconsistent associations of SB with LBP [20]. A meta-analysis by Alzahrani and colleagues reported no association of SB with the prevalence of LBP but reported positive associations with LBP intensity and disability [20]. Notwithstanding the methodological limitations that might be present in the above-mentioned reviews, a specifically clear distinction was not made between SB and physical inactivity in the inclusion criteria [19], the possibility of reverse causation within cross-sectional designs limits the comparability of our findings with these previous reviews of prospective studies. Adults, especially those with multi-morbidities including MSP conditions may often be less active and resort to SB which may have a pain modulation effect [130]. A review, for instance, had previously found that SB is much common in people with knee osteoarthritis [131]. We found that there is a positive cross-sectional association of SB with knee pain, but of limited strength due to a small number of reviewed studies; however, causal relation cannot be inferred from a cross-sectional finding with a potential reverse causation bias.

### Occupational sedentary behaviour and musculoskeletal pain conditions

Our meta-analysis of cross-sectional studies found a positive association of self-reported total workplace sitting with the risk of LBP and neck/shoulder pain. A subgroup analysis by non-tradespeople and tradespeople for the risk of LBP shows the association is significant only in the non-tradespeople. Although limited in terms of the number of studies available, our best-evidence synthesis indicates the association of device-measured workplace sitting with LBP or LBP-intensity was inconsistent in cross-sectional studies of both non-tradespeople and tradespeople but suggests a potential protective association in prospective studies which could be moderated by occupational demand. Also, there is an indication from three studies (including a prospective study) from the same dataset of a negative association of workplace sitting with neck/shoulder pain-intensity in tradespeople. Furthermore, our meta-analysis showed no association of self-reported workplace sitting with the risk of pain in extremities. Nevertheless, a subgroup analysis indicates self-reported workplace sitting may have a protective association for pain in lower limbs. Evidence of prospective associations of self-reported workplace sitting with MSP conditions is insufficient due to a limited number of reviewed prospective studies. Additionally, we observed in a meta-analysis of three cross-sectional studies on vehicle time and LBP that excessive time spent sitting in a vehicle increases the odds of LBP, yet this cross-sectional association is non-significant.

Additionally, though limited by the number of studies, computer time was found to be cross-sectionally but not prospectively associated with neck/shoulder pain in the positive direction, and there was inconclusive evidence on the direction with LBP and general MSP. Also, from the reviewed experimental/intervention studies, we observed evidence of positive associations of reduced workplace sitting with a reduction in LBP, neck/shoulder pain, and general MSP/discomfort; nevertheless, no evidence on whether reduced workplace sitting is associated with a reduction in extremities pain.

A recent review of prospective studies has reported that device-measured workplace sitting among tradespeople to be associated with a reduced risk of LBP and neck pain [26]. Compared to our review, there are some similarities in the findings even though we were limited by the volume of studies reviewed in this context. For example, there was an indication from our reviewed prospective studies that device-measured workplace sitting could have a negative association with LBP-intensity which may be dependent on the physical demand of the occupation. Similarly, there is a likelihood of a negative cross-sectional association of device-measured workplace sitting bout with LBP-intensity which is potentially moderated by overweight/obesity in tradespeople. Additionally, our reviewed studies on device-measured workplace sitting in tradespeople suggest a probable negative association with neck/shoulder pain-intensity. A possible explanation of the observed tendency of protective associations of workplace sitting with some MSP conditions in tradespeople could be the physically intensive nature of some of these occupations compared to desk-based occupations. For instance, we also observed in our meta-analysis that self-reported workplace sitting of cross-sectional studies be positively associated with LBP in non-tradespeople but not in tradespeople, albeit in a limited number of studies. Some proponents of the “physical activity paradox” assert that sitting could be of health benefit in individuals who regularly engage in high occupational physical activity as sitting may allow some form of rest and recovery [40, 41]. These indications in our review are, however, inconclusive and warrant further investigations in diverse occupational settings to ascertain these findings.

Generally, our meta-analysis of cross-sectional studies indicated that self-reported workplace sitting significantly increases the odds of LBP by 1.47 times; and was marginally higher, by 1.56 times, in a subgroup of non-tradespeople (Fig. 3). In contrast, previous reviews have reported no evidence of association of workplace sitting with LBP [22, 23]. These previous reviews included both cross-sectional and prospective studies; in contrast, our evidence was synthesised from only cross-sectional studies, therefore, limiting any interpretation of a causal relationship of workplace sitting with LBP. The possibility of reverse causation along with bias in self-reported data in the cross-sectional studies reviewed may adversely affect the quality of evidence in the observed positive association. Similarly, this may have affected the interpretation of the association between SB and neck/shoulder pain.

Also, our best evidence synthesised indicates there is moderate cross-sectional evidence that computer time (≥ 4 h/day) increases the risk of neck/shoulder pain; two previous systematic reviews of prospective studies [21, 24] and RCT studies [21], however, have reported no association of computer time with the risk of neck pain. Furthermore, there is informative evidence of a probable association between vehicle time and LBP. A pooled meta-analysis of three cross-sectional studies indicates prolonged hours of sitting in a vehicle increase the odds of LBP, but the association is not statistically significant. No published review studies, to our knowledge, have specifically investigated vehicle time and MSP conditions, nonetheless, a recent review has reported that MSP conditions are highly prevalent in vehicle drivers [132]. The cross-sectional evidence of computer and vehicle times is, however, of low quality and limited by a small volume of reviewed studies precluding the possibility of causal relationships.

Evidence on the effects of changes in workplace sitting on MSP conditions is scarce. In contrast, workplace interventions to reduce MSP conditions have provided some insight into the benefit of increased workplace physical activity on musculoskeletal health for comparison [133–136]. For instance, increased occupational physical activity is reported to be associated with reduced general MSP symptoms [133, 134, 136]. Also, a review of experimental studies has reported that device-measured continuous uninterrupted sitting is associated with the increased immediate report of LBP in adults [25]. The evidence from our review also suggests experiments/interventions that reduce total workplace sitting time or sitting bout duration potentially reduce general MSP/discomfort, especially in the lower back and the neck/shoulder. This is consistent with a review that found that workplace interventions potentially reduce LBP and neck/shoulder pain among workers [133, 134]. These findings should be treated with caution due to the limited number and variations in the reviewed experimental/intervention studies.

This review did not specifically investigate the potential mechanisms that underpin the association of occupational and non-occupational SB with MSP conditions. Nevertheless, some previous studies have speculated the potential mechanisms of the association between SB and MSP conditions such as LBP [37, 137]. For instance, studies that have investigated biomechanical and physiological mechanisms of LBP suggest occupational sitting increases spinal load and accumulation of metabolites that accelerate degenerative changes in vertebral discs [36, 37]. The available systematic review literature on the association between SB and MSP conditions is yet to address potential biological mechanisms. Nonetheless, there is an observation in this current review that indicates the association of occupational SB with, for example, LBP may be modulated by overweight/obesity. Increasingly, higher volumes of SB are linked with adiposity [38]; adipose tissue is metabolically active, releasing pro-inflammatory cytokines and adipokines that may potentiate inflammatory changes in the musculoskeletal systems leading to pain [138]. There is, therefore, a need for further studies on the potential biological mechanisms that explain the associations.

### Implications for practice and research

Despite the methodological challenges within the reviewed studies in this current systematic review, the overall observation which is supported by the evidence from experimental/intervention studies is that SB may have a detrimental association with musculoskeletal health. Theoretically, replacing a portion of time spent in SB with physical activity could beneficially impact MSP conditions. For instance, one of our reviewed studies [67] reported that substituting 30 min of a full day’s total sedentary time with 30 min of moderate-to-vigorous physical activity (MVPA) may reduce general MSP by 29%. Further, evidence from some of the reviewed experimental/intervention studies also indicates that reduced workplace sitting, and increased standing or walking did not worsen general MSP symptoms [116, 121, 123]. Current WHO physical activity and sedentary behaviour guidelines, in part, recommend reducing and interrupting prolonged SB or sitting with physical activity of any intensity for improved health outcomes [139]. This practice guideline could be encouraged in adults, especially in occupational settings to minimise the risk of MSP conditions.

Our review has identified some knowledge gaps for potential further studies. For instance, inconsistent associations were observed for self-reported and device-measured SB. The evidence of positive cross-sectional associations of SB with MSP conditions was mainly based on self-reported SB. The evidence synthesised from the few studies that investigated device-measured SB was inconsistent with MSP conditions. There is evidence of disparities in device-measured and self-reported SB in adults, with increased potential of self-reported tools to either underestimate or overestimate SB [27]. Furthermore, there were some variations in the measures of MSP conditions; some studies investigated single MSP conditions and some multiple MSP conditions, which could impact the studies' quality and their comparability. Also, the review identified insufficient evidence of prospective associations of SB with MSP conditions and could not make definite conclusions regarding possible causal relationships due to the limited number of prospective studies. Hence, future attention on the application of device-measured SB will be relevant in this context to minimise bias in the probable associations, taking into consideration the outcome measure. Specifically, future research focus could explore the use of posture-based activPAL, the gold standard instrument for measuring sitting time, in prospective study designs. Additionally, some contemporary analytical approaches in the field, such as compositional data analysis could be applied to investigate SB associations relative to other 24-h movement behaviours such as physical activity and sleep with MSP conditions [140]. This review mainly examined the associations of SB with different types of MSP conditions and did not consider the underlying pathophysiology of the MSP conditions. Future studies could also examine the direction of the associations in subgroups of particular MSP conditions. For instance, the direction of association of SB with LBP secondary to lumbar disc degeneration may contrast with the association of SB with LBP due to facet joint inflammation.

This review and previous reviews have not investigated the probable interaction of chronic diseases in the association of SB with MSP conditions. Importantly, MSP conditions are highly prevalent in the presence of multi-morbidities [3, 4], and also emerging as common comorbidities in some chronic diseases, especially type 2 diabetes (T2D) [141–143]. Evidence from an observational study, for example, suggests there is a potential interaction of SB with the association of T2D with MSP conditions in adults [141]. Therefore, it will be of great interest for potential future studies, including cross-sectional, prospective, and RCTs study designs to also focus on the interaction of some chronic diseases such as obesity, T2D, cardiovascular diseases, etc. with the association of SB with MSP conditions. Research in this direction will also provide insight into the understanding of the potential biological mechanisms of SB/MSP conditions associations.

### Strengths and limitations

A key strength of this review is its distinct consideration of occupational and non-occupational SB, as well as a wide range of MSP conditions. Also, the evidence synthesis was organised into SB domains and measures, likewise the type of MSP outcomes. For a better insight into the risk associations, studies conducted exclusively in clinical groups diagnosed with MSP conditions and those of autoimmune disease-related MSP conditions were not reviewed.

However, we acknowledge that there are some limitations, and caution should be applied when interpreting the findings. First, a single reviewer initially excluded irrelevant studies by title and abstract screening in stage one of two-phase screening; this might have contributed to exclusion of some relevant studies [144]; however, where there was uncertainty regarding inclusion, such studies were considered for second-stage screening by two independent reviewers. Second, most of the studies reviewed were cross-sectional in design, hence, causality cannot be inferred. Third, there were a limited number of studies, especially prospective and experimental/intervention studies, as well as high methodological and analytical variations in the reviewed studies. The limited number of experimental/intervention studies, especially RCTs, may be because we used the term “sitting” to search for “sitting reduction interventions” and “sitting experimental studies” instead of searching for specific interventions (e.g., sit-stand workstations, stand-up desk, etc.). Also, the limited number of prospective studies might be a result of publication bias as some prospective studies on risk factors for MSP conditions may have examined sitting as a risk factor or have accounted for SB as a confounder but found no association and did not report in the Abstract; therefore, these studies would not be identified by the search.

Fourth, a small number of studies were included in the meta-analyses to estimate the pooled effect sizes, resulting in moderate-to-high heterogeneity in some of the outputs. It is important, however, to note that the inverse-variance meta-analysis approach has a limitation of estimating a false high heterogeneity [145]. Therefore, the observed heterogeneity may be potentially due to variations within the studies but not bias in the results. Fifth, we did not consider the covariates adjusted for in the individual studies in our evidence synthesis. For instance, evidence synthesised from studies that accounted for physical activity might be different from those that did not control for physical activity in analyses. Similarly, studies that accounted for sitting positions assumed (e.g., leaning forward or backward) and occupational activities may influence the evidence synthesised from those that did not account for these factors. Also, specific sources of potential bias and specific limitations that were commented upon by the authors of the reviewed studies, or which potentially could be identified in the studies might impact the findings but were not considered in the evidence synthesis.

Sixth, strict selection criteria were adapted to enhance the efficiency of the review, however, this might consequently lead to studies with relevant information being excluded. Furthermore, we adapted the PICO format in constructing our search terms which included search terms for the outcome to maximise the search output. There is the possibility that the outcome may not be well described in the title and abstract of potential studies and therefore not indexed in databases with controlled vocabulary terms leading to missing potential studies [146]. Finally, only articles published in the English language were reviewed; this could bias our finding as informative evidence in studies published in other languages may have been missed. To minimise this shortcoming, however, we also searched grey literature to identify more relevant studies.

## Conclusions

Our systematic review identified evidence of cross-sectional associations of SB (occupational and non-occupational) with MSP conditions. The direction of the association of occupational SB with some MSP conditions, nonetheless, may be dependent on the type and physical demand of the occupation involved. The possibility of reverse causation could not, however, be discounted from the observed cross-sectional associations. Further, evidence from intervention studies shows that reducing prolonged sitting at work reduces MSP conditions and discomforts. There was, however, limited evidence of prospective associations of SB with MSP conditions. Importantly though, the review highlighted some knowledge gaps, including a limited number of studies using device-measured SB and MSP conditions, as well as limited prospective and RCT study designs. Considering the inconsistencies of the review’s findings, as well as the highlighted knowledge gaps, further research, especially prospective and RCT studies, is required to better understand the association of SB in occupational and non-occupational settings with MSP conditions. Furthermore, as studies of clinical groups with existing MSP conditions were not reviewed in this current study, future review studies could consider exclusively reviewing this study population. Such studies could also consider examining the contribution of the presence of MSP conditions to the engagement in SB. Also, there is the need for tailored studies to understand the potential interactions of chronic diseases such as obesity, T2D, and cardiovascular diseases in the association of SB with MSP conditions.

## Supplementary Information

**Additional file 1: Supplementary Table 1.** Search key terms and strings strategy. **Supplementary Table 2.** Studies excluded after full-text screening. **Supplementary Figure 1.** Full-day SB and LBP: (A) A forest plot of sensitivity analysis after excluding two studies, Kulaivelan et al. 2018 and Machado et al. 2018 from the analysis. (B) A funnel plot showing publication bias. **Supplementary Figure 2.** Self-reported workplace sitting and LBP: (A) A forest plot of sensitivity analysis after excluding two studies of lower quality assessment score, Anita et al. 2019 and van Vuuren et al. 2005 from the analysis. (B) A funnel plot showing publication bias. **Supplementary Figure 3.** Self-report workplace sitting and neck/shoulder pain: (A) A forest plot of sensitivity analysis after excluding two studies of low-quality, Cagnie et al. 2007 and Chrasakaran et al. 2003 from the analysis. (B) A funnel plot showing publication bias. **Supplementary Figure 4.** Self-reported workplace sitting and extremities pain: (A) A forest plot of sensitivity analysis after excluding two studies of low-quality, Chrasakaran et al. 2003 and Tsigonia et al. 2009 from the analysis. (B) A funnel plot showing publication bias. **Supplementary Figure 5.** Vehicle time and LBP: (A) A forest plot of sensitivity analysis after excluding the study, Hakim et al. 2018 with low-quality from the analysis. (B) A funnel plot showing publication bias.

## Data Availability

Almost all data generated or analysed during this study are included in this published article [and its supplementary information files]. Further datasets used and/or analysed during the current study are available from the corresponding author on reasonable request.

## Abbreviations

SB: Sedentary behaviour
MSP: Musculoskeletal pain
PRISMA: Preferred Reporting Items for Systematic Reviews and Meta-Analyses
CINAHL: Cumulative Index to Nursing and Allied Health Literature
AMED: Allied and Complementary Medicine Database
LBP: Lower back pain
OR: Odds ratio
GBD: Global Burden of Disease
METs: Metabolic equivalents
WHO: World Health Organisation
PICO: Population, Intervention, Control/Comparison, and Outcome
RCTs: Randomized controlled trials
BMI: Body mass index
QualSyst: Standard Quality Assessment Criteria for Evaluating Primary Research Papers from a Variety of Fields
N/A: Not applicable
UBP: Upper back pain
RevMan5: Review Manager version 5.4.1
USA: United States of America
UK: United Kingdom
TV: Television-viewing
DPHACTO: Danish PHysical ACTivity cohort with Objective measurements
MVPA: Moderate-to-vigorous physical activity
T2D: Type 2 diabetes
