# Supplementary material for: Musculoskeletal pain and sedentary behaviour in occupational and non-occupational settings: a systematic review with meta-analysis
Source: Int J Behav Nutr Phys Act. 2021 Dec 13;18:159. doi: 10.1186/s12966-021-01191-y (PMC8666269; doi:10.1186/s12966-021-01191-y)
Supplement: Supplementary file 1 — Additional file 1: Supplementary Table 1. Search key terms and strings strategy. Supplementary Table 2. Studies excluded after full-text screening. Supplementary Figure 1. Full-day SB and LBP: (A) A forest plot of sensitivity analysis after excluding two studies, Kulaivelan et al. 2018 and Machado et al. 2018 from the analysis. (B) A funnel plot showing publication bias. Supplementary Figure 2. Self-reported workplace sitting and LBP: (A) A forest plot of sensitivity analysis after excluding two studies of lower quality assessment score, Anita et al. 2019 and van Vuuren et al. 2005 from the analysis. (B) A funnel plot showing publication bias. Supplementary Figure 3. Self-report workplace sitting and neck/shoulder pain: (A) A forest plot of sensitivity analysis after excluding two studies of low-quality, Cagnie et al. 2007 and Chrasakaran et al. 2003 from the analysis. (B) A funnel plot showing publication bias. Supplementary Figure 4. Self-reported workplace sitting and extremities pain: (A) A forest plot of sensitivity analysis after excluding two studies of low-quality, Chrasakaran et al. 2003 and Tsigonia et al. 2009 from the analysis. (B) A funnel plot showing publication bias. Supplementary Figure 5. Vehicle time and LBP: (A) A forest plot of sensitivity analysis after excluding the study, Hakim et al. 2018 with low-quality from the analysis. (B) A funnel plot showing publication bias. [file 12966_2021_1191_MOESM1_ESM.docx]

**SUPPLEMENTARY FILES**

**Supplementary Table 1:** Search key terms and strings strategy

Sample Medline database search syntax for the review’s study search. The search strings and Boolean operators’ construction was replicated across the other selected databases.

**MH = Medline Subject Headlines TI = Title AB = Abstract**

| **Search** | **Search Term** | **Search Options** | **Result** |
| --- | --- | --- | --- |
| **EXPOSURE** | | | |
| S1 | (MH "Sedentary Behavior") OR (MH "Sitting Position") OR (MH "Screen Time") | Expanders - Apply equivalent subjects  Search modes - Find all my search terms |  |
| S2 | TI ( “Sedentary behavio*” OR “Sedentary lifestyle” OR Sedenta* OR sitting OR “prolong* sitting” OR "uninterrupted sitting” OR “Sitting time” OR “Screen time” OR “Sitting position” OR “sitting disease*” OR "television viewing time" OR "television-viewing time" OR "TV viewing time" OR "TV-viewing time") OR AB (“Sedentary behavio*” OR “Sedentary lifestyle” OR Sedenta* OR sitting OR “prolong* sitting” OR "uninterrupted sitting” OR “Sitting time” OR “Screen time” OR “Sitting position” OR “sitting disease*” OR "television viewing time" OR "television-viewing time" OR "TV viewing time" OR "TV-viewing time") | Expanders - Apply equivalent subjects  Search modes - Find all my search terms |  |
| S3 | S1 OR S2 | Expanders - Apply equivalent subjects  Search modes - Find all my search terms |  |
| **OUTCOME** | | | |
| S4 | (MH "Musculoskeletal Pain+") OR (MH "Musculoskeletal Diseases+") OR (MH "Neck Pain") OR (MH "Arthritis+") OR (MH "Arthritis, Rheumatoid+") OR (MH "Arthritis, Psoriatic+") OR (MH "Arthritis, Gouty+") OR (MH "Crystal Arthropathies+") OR (MH "Osteoarthritis+") OR (MH "Osteoarthritis, Hip+") OR (MH "Osteoarthritis, Knee+") OR (MH "Osteoarthritis, Spine+") OR (MH "Back Pain+") OR (MH "Low Back Pain+") OR (MH "Carpal Tunnel Syndrome+") OR (MH "Tarsal Tunnel Syndrome+") OR (MH "Bursitis+") OR (MH "Dupuytren Contracture+") OR OR (MH "Tenosynovitis+") OR (MH "Spondylitis+") OR (MH "Spondylitis, Ankylosing+") OR (MH "Spondylarthropathies+") OR (MH "Spondylarthritis+") OR (MH "Joint Diseases+") OR (MH "Arthropathy, Neurogenic+") OR (MH "Diabetic Neuropathies+") OR (MH "Hyperostosis, Diffuse Idiopathic Skeletal+") OR (MH "Enthesopathy+") OR (MH "Fibromyalgia+") | Expanders - Apply equivalent subjects  Search modes - Find all my search terms |  |
| S5 | TI ( “Musculoskeletal pain disorder*” OR “Musculoskeletal pain*” OR “Musculoskeletal disorder*” OR “Musculoskeletal system disorder*” OR “osteomuscular disease*” OR “osteomuscular disorder*” OR “osteomuscular pain*” OR osteoarthritis OR osteochondritis OR arthritis OR polyarthritis OR capsulitis OR spondylitis OR arthropath* OR “shoulder pain*” OR “knee pain*” OR “back pain*” OR “lumbar pain*” OR “neck pain*” OR “cervical pain*” OR “joint pain*” OR "ankle pain*" OR “rheumatoid arthritis” OR gout* OR “limited joint mobility syndrome” OR “diabetic cheiroarthropathy” OR cheiroarthropathy OR “carpal tunnel syndrome” OR “Dupuytren* contracture” OR “Dupuytren* disease*” OR “stiff hand syndrome” OR “flexor tenosynovitis” OR “Charcot osteoarthropathy” OR “neuropathic arthropathy” OR “diabetic muscular infarction” OR “proximal motor neuropathy” OR “acute proximal neuropathy” OR “diffuse idiopathic skeletal hyperostosis syndrome” OR “DISH syndrome” OR enthesopathy OR fibromyalgia OR “fibromyalgia Syndrome” OR FMS OR “Tarsal Tunnel Syndrome”) OR AB (“Musculoskeletal pain disorder*” OR “Musculoskeletal pain*” OR “Musculoskeletal disorder*” OR “Musculoskeletal system disorder*” OR “osteomuscular disease*” OR “osteomuscular disorder*” OR “osteomuscular pain*” OR osteoarthritis OR arthritis OR polyarthritis OR capsulitis OR spondylitis OR arthropath* OR “shoulder pain*” OR “knee pain*” OR “back pain*” OR “lumbar pain*” OR “neck pain*” OR “cervical pain*” OR “joint pain*” OR "ankle pain*" OR "leg pain*" OR “rheumatoid arthritis” OR gout* OR “limited joint mobility syndrome” OR “diabetic cheiroarthropathy” OR cheiroarthropathy OR “carpal tunnel syndrome” OR “Dupuytren* contracture” OR “Dupuytren* disease*” OR “stiff hand syndrome” OR “flexor tenosynovitis” OR “Charcot osteoarthropathy” OR “neuropathic arthropathy” OR “diabetic muscular infarction” OR “proximal motor neuropathy” OR “acute proximal neuropathy” OR “diffuse idiopathic skeletal hyperostosis syndrome” OR “DISH syndrome” OR enthesopathy OR fibromyalgia OR “fibromyalgia Syndrome” OR FMS OR “Tarsal Tunnel Syndrome”) | Expanders - Apply equivalent subjects  Search modes - Find all my search terms |  |
| S6 | S4 OR S5 | Expanders - Apply equivalent subjects  Search modes - Find all my search terms |  |
| **POPULATION AND COMPARISON** | | | |
| S7 | (MH "Adult+") OR (MH "Young Adult") OR (MH "Frail Elderly") OR (MH "Aged+") | Expanders - Apply equivalent subjects  Search modes - Find all my search terms |  |
| S8 | TI ( Adult* OR “young adult*” OR “middle-aged adult*” OR “middle aged adult*” OR “older adult*” OR Flail adult* OR “flail older adult*” OR Elderly OR aged ) OR AB ( Adult* OR “young adult*” OR “middle-aged adult*” OR “middle aged adult*” OR “older adult*” OR Flail adult* OR “flail older adult*” OR Elderly OR aged ) NOT ( child* OR adolescen* OR "adolescen* age*" OR Teenag* OR "Teenag* age*") | Expanders - Apply equivalent subjects; Apply related words  Search modes - Find all my search terms |  |
| S9 | S7 OR S8 | Expanders - Apply equivalent subjects  Search modes - Find all my search terms |  |
| **Studies Identified** | | | |
| **S13** | **S3 AND S6 AND S9** | Limiters - Date of Publication: 20000101-20201101  Expanders - Apply equivalent subjects  Search modes - Find all my search terms |  |

**Supplementary Table 2:** Studies excluded after full-text screening

| **No.** | **Study ID** | **Title** | **Reason for Exclusion** |
| --- | --- | --- | --- |
| 1 | Adeyemi et al. 2019 | The effect of furniture intervention on the occurrence of musculoskeletal disorders and academic performance of students in North-West Nigeria | Wrong exposure; No exposure was measured |
| 2 | Alipour et al. 2008 | Occupational neck and shoulder pain among automobile manufacturing workers in Iran | Wrong exposure; No clear SB measure was described |
| 3 | Alonso Monteiro Bezerra et al. 2018 | Prevalence of chronic musculoskeletal conditions and associated factors in Brazilian adults - National Health Survey | Wrong exposure; No clear SB measure was described |
| 4 | Amorim et al. 2017 | Does sedentary behavior increase the risk of low back pain? A population-based co-twin study of Spanish twins | Excluded after discussion with senior reviewers - Wrong exposure |
| 5 | Andrade-Gómez et al. 2017 | Watching TV has a distinct sociodemographic and lifestyle profile compared with other sedentary behaviors: A nationwide population-based study | Excluded after discussion with senior reviewers - Specifically investigated SB |
| 6 | Arheiam & Ingafou, 2015 | Self-reported occupational health problems among Libyan dentists | Wrong exposure; No clear SB measure was described |
| 7 | Aytutuldu et al. 2020 | Musculoskeletal pain and its relation to individual and work-related factors: A cross-sectional study among Turkish office workers who work using computers | Wrong exposure; No clear measure of exposure |
| 8 | Babar-Craig et al. 2003 | Prevalence of back and neck pain amongst ENT consultants: national survey | Wrong exposure; No clear SB measure was described |
| 9 | Babiolakis et al. 2014 | Differences in lumbopelvic control and occupational behaviours in female nurses with and without a recent history of low back pain due to back injury | No relationship between SB and MSP conditions reported; Include population with existing MSP condition |
| 10 | Backåberg et al. 2014 | Impact of musculoskeletal symptoms on general physical activity during nursing education | Wrong exposure; No relationship between SB and MSP conditions was reported |
| 11 | Badley & Ansari, 2010 | Arthritis and arthritis-attributable activity limitations in the United States and Canada: a cross-border comparison | Wrong exposure; No SB measure only stated "less active", "low PA", "inadequate PA", "inactive", "PA inactivity" |
| 12 | Badley & Perruccio, 2017 | Population-Based Study of Changes in Arthritis Prevalence and Arthritis Risk Factors Over Time: Generational Differences and the Role of Obesity | Discussed - Focused on birth cohort effect as exposure |
| 13 | Balogh et al. 2004 | Self-assessed and directly measured occupational physical activities--influence of musculoskeletal complaints, age and gender | Wrong exposure and outcome relation described |
| 14 | Banerjee et al. 2016 | Work Related Musculoskeletal Morbidity among Tailors: A Cross Sectional Study in a Slum of Kolkata | Wrong exposure; No clear SB measure was described |
| 15 | Barone Gibbs et al. 2018 | Reducing sedentary behaviour to decrease chronic low back pain: the stand back randomised trial | Wrong population; Population with existing MSP condition |
| 16 | Bau et al. 2017 | Correlations of Neck/Shoulder Perfusion Characteristics and Pain Symptoms of the Female Office Workers with Sedentary Lifestyle | Wrong exposure; No SB measure only stated "less active" |
| 17 | Bell et al. 2020 | Physical activity and sedentary behaviour in people with inflammatory joint disease: a cross sectional study | Population with existing MSP conditions; Autoimmune-related MSP conditions |
| 18 | Bláfoss et al. 2020 | Is hard physical work in the early working life associated with back pain later in life? A cross-sectional study among 5700 older workers | Wrong exposure; No clear relation between SB and MSP condition reported |
| 19 | Blümel et al. 2017 | Obesity is associated with a higher prevalence of musculoskeletal pain in middle-aged women | Wrong exposure; No standard SB measure - not engage PA |
| 20 | Bohman et al. 2013 | The influence of self-reported leisure time physical activity and the body mass index on recovery from persistent back pain among men and women: a population-based cohort study | Wrong exposure; Population with existing MSP condition |
| 21 | Brown et al. 2000 | Leisure time physical activity in Australian women: relationship with well being and symptoms | Wrong exposure; No clear SB measure was described |
| 22 | Bruce et al. 2005 | Aerobic exercise and its impact on musculoskeletal pain in older adults: a 14 year prospective, longitudinal study | Wrong exposure; No clear SB measure was described |
| 23 | Centers for Disease Control; Prevention (CDC) et al. 2011 | Arthritis as a potential barrier to physical activity among adults with obesity--United States, 2007 and 2009 | Wrong outcome; Wrong exposure; No clear SB measure described |
| 24 | Centers for Disease Control; Prevention (CDC) et al. 2011 | State-specific prevalence of no leisure-time physical activity among adults with and without doctor-diagnosed arthritis--United States, 2009 | Wrong outcome; Wrong exposure; No clear SB measure described |
| 25 | Chahbi et al. 2018 | The prevalence of painful diabetic neuropathy in 300 Moroccan diabetics | Wrong outcome; No exposure described |
| 26 | Chi & Lin, 2008 | An ergonomic evaluation of a call center performed by disabled agents | Wrong exposure; No clear SB measure was described |
| 27 | Citko et al. 2018 | Sedentary Lifestyle and Nonspecific Low Back Pain in Medical Personnel in North-East Poland | Wrong exposure; No exposure was measured |
| 28 | Dagne et al. 2020 | Work-related musculoskeletal disorders and associated factors among bank workers in Addis Ababa, Ethiopia: a cross-sectional study | Discussed - No exposure MSP condition relationship described |
| 29 | De Albuquerque, 2012 | The relationship between the lumbar lordosis, body fat percentage, lumbar spine range of motion, physical activity level and the incidence of low back pain in females | Wrong exposure; SB or sitting time not measured |
| 30 | del Pozo-Cruz et al. 2013 | Musculoskeletal fitness and health-related quality of life characteristics among sedentary office workers affected by sub-acute, non-specific low back pain: a cross-sectional study | Wrong exposure; Population with existing LBP; SB or sitting time not measured |
| 31 | Derakhshanrad et al. 2020 | Neck pain associated with smartphone overuse: cross-sectional report of a cohort study among office workers | Wrong exposure; Exposure not measured |
| 32 | d'Errico et al. 2010 | Risk factors for upper extremity musculoskeletal symptoms among call center employees | Wrong exposure; No clear SB measure was described |
| 33 | Ding et al. 2020 | It is time to have a rest: How do break types affect muscular activity and perceived discomfort during prolonged sitting work | Discussed - No exposure MSP condition relationship described |
| 34 | Ekblom-Bak et al. 2020 | Latent profile analysis patterns of exercise, sitting and fitness in adults – Associations with metabolic risk factors, perceived health, and perceived symptoms | Wrong exposure; Wrong outcome investigated |
| 35 | Gupta et al. 2018 | Is self-reported time spent sedentary and in physical activity differentially biased by age, gender, body mass index, and low-back pain? | Wrong outcome; Specifically investigated SB as an outcome |
| 36 | Hashem et al. 2018 | Exploration of the Inter-Relationships Between Obesity, Physical Inactivity, Inflammation, and Low Back Pain | Wrong exposure; Population with existing LBP; SB or sitting time not measured |
| 37 | Heesch et al. 2007 | Relationship between physical activity and stiff or painful joints in mid-aged women and older women: a 3-year prospective study | Wrong exposure; No SB measure only stated "less active", "low PA", "inadequate PA" |
| 38 | Heneghan et al. 2018 | What is the effect of prolonged sitting and physical activity on thoracic spine mobility? An observational study of young adults in a UK university setting | Discussed - Wrong outcome |
| 39 | Heneweer & Vanhees, 2009 | Physical activity and low back pain: a U-shaped relation? | Wrong exposure; No clear SB measure was described |
| 40 | Henriques et al. 2020 | Low back pain among hospital nursing assistants | Wrong exposure; No exposure MSP condition relationship described |
| 41 | Holmberg & Thelin, 2010 | Predictors of sick leave owing to neck or low back pain: a 12-year longitudinal cohort study in a rural male population | Wrong outcome; Wrong exposure; Population with existing MSP condition |
| 42 | Husemann et al. 2009 | Comparisons of musculoskeletal complaints and data entry between a sitting and a sit-stand workstation paradigm | Excluded after discussion with senior reviewers - Wrong outcome; No clear SB measure described |
| 43 | Inoue et al. 2015 | The prevalence and characteristics of low back pain among sitting workers in a Japanese manufacturing company | Wrong exposure; No clear SB measure was described |
| 44 | Jiménez-Sánchez et al. 2010 | Has the prevalence of invalidating musculoskeletal pain changed over the last 15 years (1993-2006)? A Spanish population-based survey | Wrong exposure; Wrong population - included population aged from 16 years and above |
| 45 | Johnston et al. 2019 | Feasibility and impact of sit-stand workstations with and without exercise in office workers at risk of low back pain: A pilot comparative effectiveness trial | Discussed - Wrong exposure-outcome relation - focused on standing |
| 46 | Jonsdottir et al. 2019 | Factors associated with chronic and acute back pain in Wales, a cross-sectional study | Wrong exposure; Wrong population; No clear SB measure described |
| 47 | Kaartinen, 2020 | Diversity of sport activities, leisure-time physical activity, and spinal pain: A Finnish Twin Study | Wrong exposure; No exposure was measured |
| 48 | Kar & Hedge, 2020 | Effect of workstation configuration on musculoskeletal discomfort, productivity, postural risks, and perceived fatigue in a sit-stand-walk intervention for computer-based work | Wrong exposure; No exposure was measured |
| 49 | Kayihan, 2014 | Relationship between daily physical activity level and low back pain in young, female desk-job workers | Wrong exposure; No SB measure only stated "less active", "low PA", "inadequate PA" |
| 50 | Khruakhorn et al. 2010 | Prevalence and risk factors of low back pain among the university staff | Excluded after discussion with senior reviewers -Wrong exposure; SB not clearly defined |
| 51 | Kingsbury et al. 2020 | Association between daily level of objective physical activity and C-Reactive protein in a representative national sample of adults with self-reported diagnosed arthritis or fibromyalgia | Wrong population; No SB and MSP condition relationship investigated; Wrong exposure and outcome relation investigated |
| 52 | Klussmann et al. 2008 | Musculoskeletal symptoms of the upper extremities and the neck: a cross-sectional study on prevalence and symptom-predicting factors at visual display terminal (VDT) workstations | Wrong exposure; No clear SB measure was described |
| 53 | Leung et al. 2018 | Duration of physical activity, sitting, sleep and the risk of total knee replacement among Chinese in Singapore, the Singapore Chinese Health Study | Discussed - Wrong outcome |
| 54 | Leveille et al. 2003 | The impact of chronic musculoskeletal pain on exercise attitudes, self-efficacy, and physical activity | Wrong exposure; No clear SB measure described; Focused on birth cohort effect |
| 55 | Mahdavi et al. 2020 | Impact of sedentary behavior on bodily pain while staying at home in COVID-19 pandemic and potential preventive strategies | Commentary; Wrong study design |
| 56 | Maniam et al. 2020 | The mapping evidences of incidence, prevalence and risk factors of chronic low back pain among adults across 3 continents | Full-text not accessible |
| 57 | Matikainen & Sjögren, 2019 | Musculoskeletal disorders and disability among forest industry workers in lower and higher sickness absence groups: a case-control study | Wrong exposure; No clear measure of exposure |
| 58 | Mazzotta et al. 2018 | Usage of Sit-Stand Workstations and Associations Between Work and Nonwork Sitting Time: An Observational Study | Discussed - Wrong outcome |
| 59 | McDonald & Salisbury, 2019 | Physical Activity, Exercise, and Musculoskeletal Disorders in Sonographers | No relationship between SB and MSP conditions reported |
| 60 | Moczek et al. 2018 | The relationship between physical activity and the prevalence of disabilities caused by back pain in men over 60 years of age | Wrong exposure; No relationship between SB and MSP conditions was reported |
| 61 | Mohseni et al. 2014 | Occupational low back pain in primary and high school teachers: prevalence and associated factors | Discussed - Wrong exposure |
| 62 | Molsted et al. 2012 | Musculoskeletal pain in patients with type 2 diabetes | Excluded after discussion with senior reviewers - Wrong exposure |
| 63 | Montakarn & Nuttika, 2016 | Physical activity levels and prevalence of low back pain in Thai call-center operators | Wrong exposure; No clear SB measure was described |
| 64 | Moreira-Silva et al. 2019 | Prevalence of Musculoskeletal Symptoms in Blue-Collar Workers: Association with Gender and Physical Activity Level | Wrong exposure; No SB measure only stated "less active", "low PA", "inadequate PA" |
| 65 | Moroder et al. 2011 | Low back pain among medical students | Discussed - No relationship between SB and MSPDs reported |
| 66 | Najafi et al. 2010 | Importance of time spent standing for those at risk of diabetic foot ulceration | Wrong exposure and outcome; Population with existing MSP condition |
| 67 | Nelson-Wong et al. 2020 | Increasing standing tolerance in office workers with standing-induced back pain | Wrong population; Population with existing MSP condition |
| 68 | Nijs et al. 2019 | Lifestyle and chronic pain across the lifespan: an inconvenient truth? | Commentary/review; Wrong publication |
| 69 | Nourbakhsh et al. 2001 | Effects of lifestyle and work-related physical activity on the degree of lumbar lordosis and chronic low back pain in a Middle East population | Wrong exposure; No clear SB measure described; Population with existing MSP condition |
| 70 | Oka et al. 2020 | Association of objectively measured physical activity with combined bilateral knee and low-back pain in older adults with knee osteoarthritis: A cross-sectional study | Discussed - Population with existing MSP condition |
| 71 | Oliveira Dantas & de Lima, 2014 | The relationship between physical load and musculoskeletal complaints among Brazilian dentists | Wrong exposure; No clear SB measure was described |
| 72 | Omokhodion et al. 2000 | Prevalence of low back pain among staff in a rural hospital in Nigeria | Wrong exposure; No clear SB measure was described |
| 73 | oval-Rosario et al. 2018 | Prevalence of Arthritis Among Adults with Prediabetes and Arthritis-Specific Barriers to Important Interventions for Prediabetes - United States, 2009-2016 | Wrong exposure; No clear SB measure was described |
| 74 | Palmlöf et al. 2016 | The impact of work related physical activity and leisure physical activity on the risk and prognosis of neck pain - a population based cohort study on workers | Excluded after discussion with senior reviewers - Wrong exposure; Focus on PA |
| 75 | Perry et al. 2020 | Occupation and risk of knee osteoarthritis and knee replacement: A longitudinal, multiple-cohort study | Wrong exposure; No clear measure of exposure |
| 76 | Petersson & Abbott, 2020 | Lumbar interspinous pressure pain threshold values for healthy young men and women and the effect of prolonged fully flexed lumbar sitting posture: An observational study | Wrong exposure; Wrong exposure/exposure not clearly described |
| 77 | Porter & Gyi, 2002 | The prevalence of musculoskeletal troubles among car drivers | Discussed - Exposure and outcome relationship not reported |
| 78 | Pradeepkumar et al. 2020 | Prevalence of work related musculoskeletal disorders among occupational bus drivers of Karnataka, South India | Discussed - Wrong exposure |
| 79 | Pugh et al. 2020 | Changes in exercise and musculoskeletal symptoms of novice nurses: A one-year follow-up study | Wrong exposure; No exposure MSPD relationship described |
| 80 | Rahmani et al. 2013 | Work related neck pain in Iranian dentists: An epidemiological study | Wrong exposure; No relationship between SB and MSP conditions reported |
| 81 | Rasim et al. 2017 | Frequency and associated risk factors for neck pain among software engineers in Karachi, Pakistan | Wrong exposure; No SB measure only stated "less active", "low PA", "inadequate PA" |
| 82 | Sanya & Ogwumike, 2005 | Low back pain prevalence amongst industrial workers in the private sector in Oyo State, Nigeria | Full-text not accessible |
| 83 | Senosy et al. 2020 | Profession-related musculoskeletal disorders among Egyptian physicians and dentists | Discussed - No exposure MSP condition relationship described |
| 84 | Serranheira et al. 2020 | Low Back Pain (LBP), work and absenteeism | Discussed - No clear measure of exposure |
| 85 | Shin & Yoo, 2020 | Lumbar Movement Dysfunction Based on Movement Control Impairment Classification System in Those Who Do and Do Not Develop Transient Low Back Pain During Prolonged Sitting | Wrong exposure; No SB and MSP relation investigated |
| 86 | Sitthipornvorakul et al. 2014 | The effect of daily walking steps on preventing neck and low back pain in sedentary workers: a 1-year prospective cohort study | Wrong exposure; No clear SB measure described; No relationship between SB and MSP conditions reported |
| 87 | Solovev et al. 2020 | Total physical activity and risk of chronic low back and knee pain in middle-aged and elderly Japanese people: The Murakami cohort study | Wrong exposure; Focus on PA |
| 88 | Tagliaferri et al. 2019 | Testing the deconditioning hypothesis of low back pain: A study in 1182 older women | Wrong exposure; No clear measure of exposure |
| 89 | Tahir, 2016 | Relationship between pedometer-determined physical activity and low back pain in middle aged Finnish population (30-45):" The Young Finns Study" | Focus on PA; Wrong exposure; No relationship between SB and MSP conditions reported |
| 90 | Teichtahl et al. 2015 | Physical inactivity is associated with narrower lumbar intervertebral discs, high fat content of paraspinal muscles and low back pain and disability | Wrong exposure; No SB measure only stated "less active", "low PA", "inadequate PA" |
| 91 | Toprak et al. 2020 | Coronaphobia, musculoskeletal pain, and sleep quality in stay-at home and continued-working persons during the 3-month Covid-19 pandemic lockdown in Turkey | Wrong exposure; No SB and MSP relation investigated |
| 92 | van Gool et al. 2007 | Chronic disease and lifestyle transitions: results from the Longitudinal Aging Study Amsterdam | Wrong exposure; Wrong population; No SB measure only stated "less active", "low PA", "inadequate PA" |
| 93 | van Sloten et al. 2011 | Peripheral neuropathy, decreased muscle strength and obesity are strongly associated with walking in persons with type 2 diabetes without manifest mobility limitations | Wrong exposure; Focus on PA - steps; No clear SB measure described |
| 94 | Vancampfort et al. 2018 | Correlates of sedentary behavior in 2,375 people with depression from 6 low- and middle-income countries | Wrong outcome; Wrong outcome investigated |
| 95 | Vibha et al. 2018 | Community based study to assess the prevalence of diabetic foot syndrome and associated risk factors among people with diabetes mellitus | Wrong outcome; Wrong exposure |
| 96 | Vinaya Swetha et al. 2020 | Association between computer work and musculoskeletal disorders of neck and upper extremity in young and adult population-a survey based analysis | Full text not accessible |
| 97 | Vindigni et al. 2005 | Low back pain risk factors in a large rural Australian Aboriginal community. An opportunity for managing co-morbidities? | Discussed – Wrong exposure measure |
| 98 | Vujcic et al. 2018 | Low Back Pain among Medical Students in Belgrade (Serbia): A Cross-Sectional Study | Discussed - No relationship between SB and MSP conditions reported |
| 99 | Waller et al. 2019 | Associations of physical activity or sedentary behaviour with pain sensitivity in young adults of the Raine Study | Discussed - Wrong outcome |
| PA - Physical activity; SB - Sedentary behaviour; MSP - Musculoskeletal pain  Reasons for studies’ exclusion by the two reviewers; discrepancies were discussed among the two reviewers or with the senior reviewers before excluding those studies. | | | |

**Supplementary Figures:** Forest plot for sensitivity analysis and funnel plot for publication bias

Full-day SB and LBP

1. Forest plot


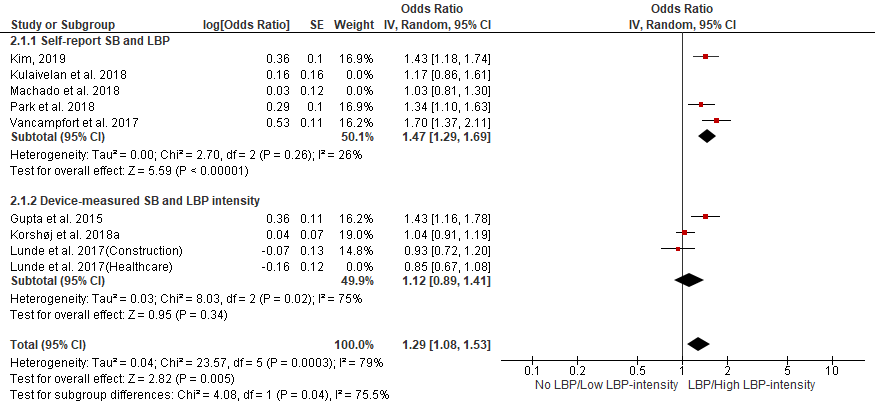


1. Funnel plot


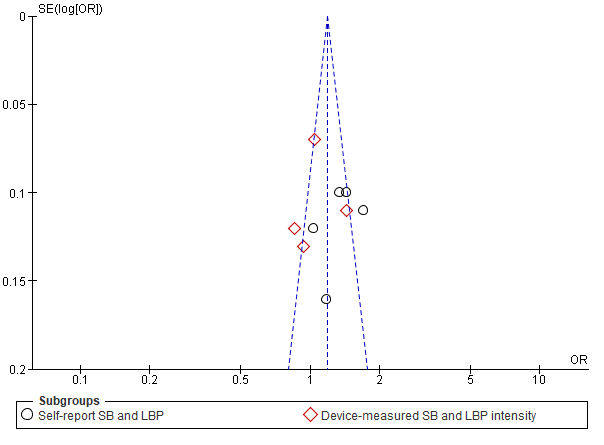


**Supplementary figure 1:** (A) A forest plot of sensitivity analysis after excluding two studies, Kulaivelan et al. 2018 and Machado et al. 2018 from the analysis. (B) A funnel plot showing publication bias.


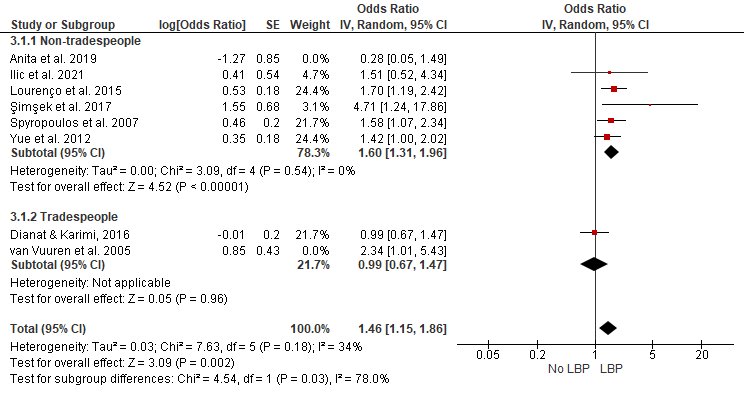
Self-reported workplace sitting and LBP

1. Forest plot
2.
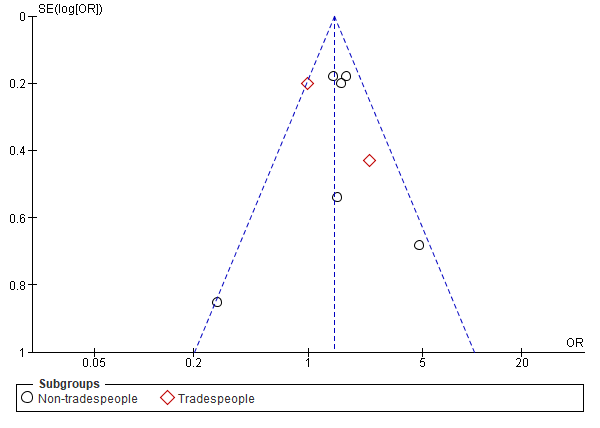
Funnel plot

**Supplementary Figure 2:** (A) A forest plot of sensitivity analysis after excluding two studies of lower quality assessment score, Anita et al. 2019 and van Vuuren et al. 2005 from the analysis. (B) A funnel plot showing publication bias.

Self-report workplace sitting and neck/shoulder pain

1. Forest plot


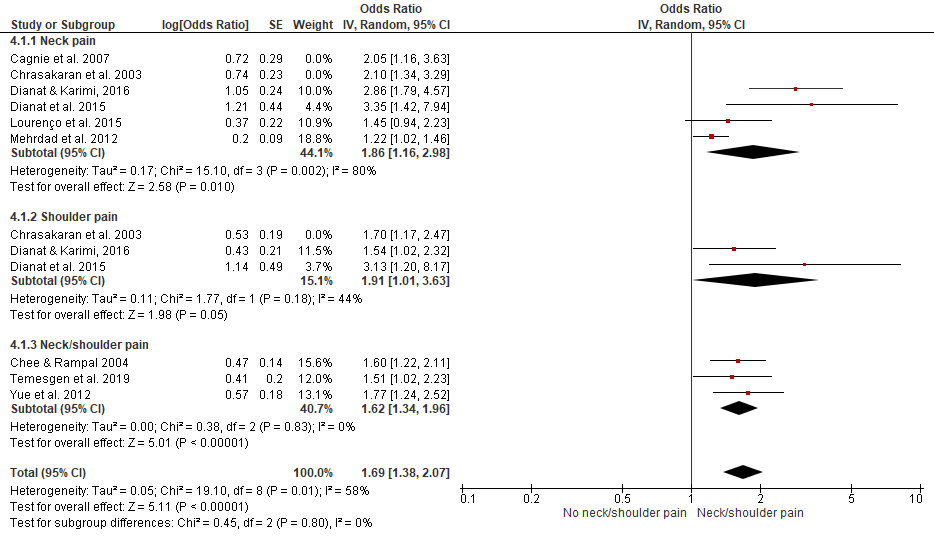


1. Funnel plot


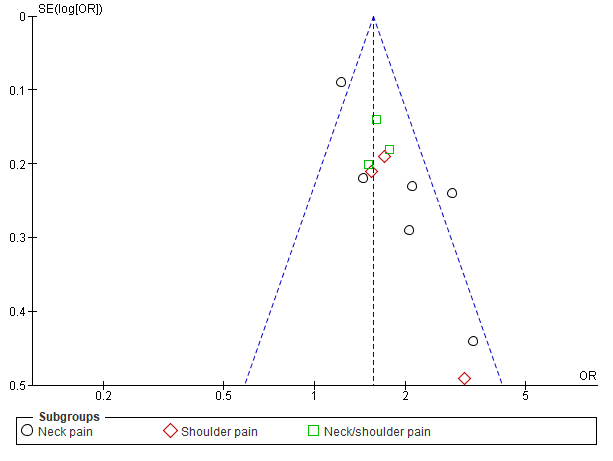


**Supplementary Figure 3:** (A) A forest plot of sensitivity analysis after excluding two studies of low-quality, Cagnie et al. 2007 and Chrasakaran et al. 2003 from the analysis. (B) A funnel plot showing publication bias.

Self-reported workplace sitting and extremities pain

1. Forest plot


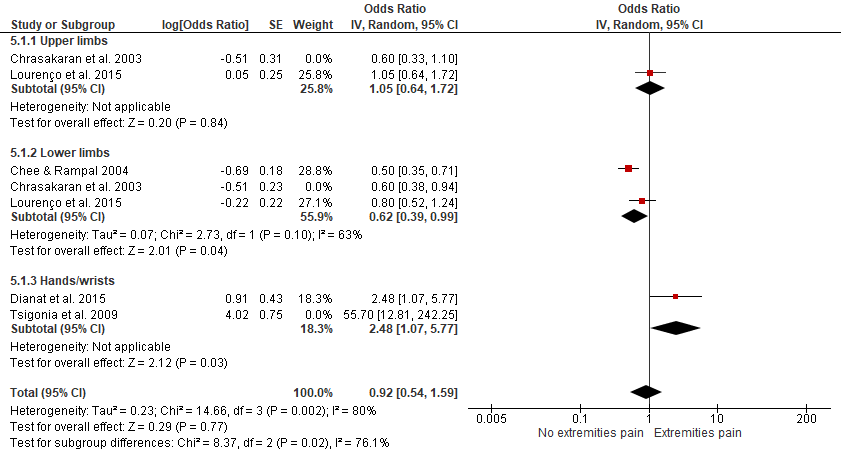


1. Funnel plot


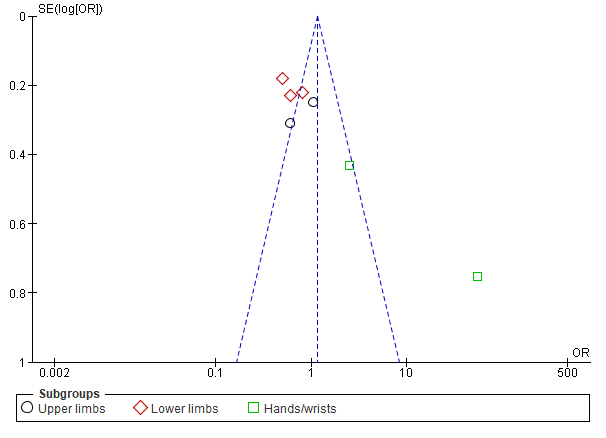


**Supplementary Figure 4:** (A) A forest plot of sensitivity analysis after excluding two studies of low-quality, Chrasakaran et al. 2003 and Tsigonia et al. 2009 from the analysis. (B) A funnel plot showing publication bias.

Vehicle time and LBP

1.
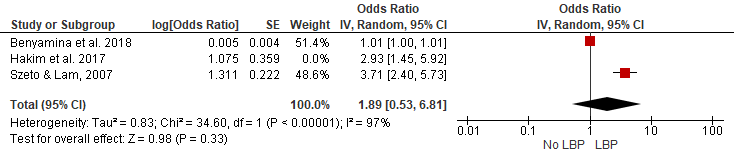
Forest plot
2. Funnel plot


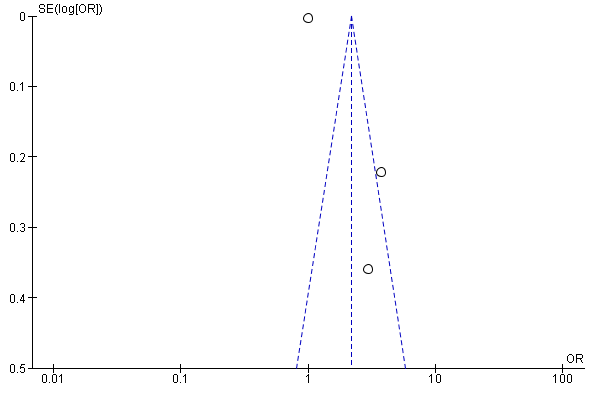


**Supplementary Figure 5:** (A) A forest plot of sensitivity analysis after excluding the study, Hakim et al. 2018 with low-quality from the analysis. (B) A funnel plot showing publication bias.
